# Auditory selective attention under working memory load

**DOI:** 10.1007/s00426-020-01437-7

**Published:** 2020-11-04

**Authors:** Rena Bayramova, Enrico Toffalini, Mario Bonato, Massimo Grassi

**Affiliations:** Department of General Psychology, University of Padova, Via Venezia, 8, Padua, Italy

## Abstract

Can cognitive load enhance concentration on task-relevant information and help filter out distractors? Most of the prior research in the area of selective attention has focused on visual attention or cross-modal distraction and has yielded controversial results. Here, we studied whether working memory load can facilitate selective attention when both target and distractor stimuli are auditory. We used a letter *n*-back task with four levels of working memory load and two levels of distraction: congruent and incongruent distractors. This combination of updating and inhibition tasks allowed us to manipulate working memory load within the selective attention task. Participants sat in front of three loudspeakers and were asked to attend to the letter presented from the central loudspeaker while ignoring that presented from the flanking ones (spoken by a different person), which could be the same letter as the central one (congruent) or a different (incongruent) letter. Their task was to respond whether or not the central letter matched the letter presented *n* (0, 1, 2, or 3) trials back. Distraction was measured in terms of the difference in reaction time and accuracy on trials with incongruent versus congruent flankers. We found reduced interference from incongruent flankers in 2- and 3-back conditions compared to 0- and 1-back conditions, whereby higher working memory load almost negated the effect of incongruent flankers. These results suggest that high load on verbal working memory can facilitate inhibition of distractors in the auditory domain rather than make it more difficult as sometimes claimed.

**Electronic supplementary material:**

The online version of this article (10.1007/s00426-020-01437-7) contains supplementary material, which is available to authorized users.

## Introduction

Humans have a remarkable ability of selectively attending to certain stimuli in noisy environments and filtering out irrelevant information. However, this ability does not always function perfectly and some circumstances lead to higher distraction than others. One of the factors that has been thought to determine the degree of distraction is cognitive load. Findings on distractor processing under cognitive load are contrasting, with evidence both for increased (Lavie et al., 2004; Lavie & De Fockert, 2005; Dalton, Santangelo, & Spence, 2009) and reduced distraction under load (Berti & Schröger, 2003; Kim, Kim, & Chun, 2005; SanMiguel, Corral, & Escera, 2008; Sörqvist & Marsh, 2015). While most of the research focuses on either intramodal visual tasks or cross-modal distraction, less is known about *auditory* selective attention under cognitive load, and consequently, much fewer paradigms have been tested. The present study adopted a novel paradigm previously used in the visual domain (Scharinger, Soutschek, Schubert, & Gerjets, 2015) to investigate the relationship between working memory-related task difficulty and distractibility in the auditory domain. We also explored a potential interaction with working memory capacity (WMC) and hypothesize on the role of individual differences in WMC in the relationship between working memory load and selective attention.

### Load theory of selective attention: cognitive control

According to load theory, interference from distractors increases when working memory is loaded (De Fockert, Rees, Frith, & Lavie, 2001; Lavie, 2000; Lavie et al., 2004). These authors suggest that cognitive control resources become depleted in high working memory load conditions and attention can ‘spill over’ distractors (Lavie et al., 2004). It is further argued that performance deteriorates under working memory load only in the presence of a salient distractor. Saliency is assumed to be given by its similarity with the target, for example, an odd color singleton distractor in shape-based search tasks (Lavie, 2005; Lavie & De Fockert, 2005; Lavie & De Fockert, 2006). No such response competition occurs when distractors are clearly unrelated to the task at hand (Logan, 1978; Woodman et al., 2001).

The principal limitation of the theory is that it does not consider the contexts where cognitive load can facilitate selective attention. For example, Kim et al. (2005) emphasized the detrimental effect of the overlap between the stimuli used for loading working memory and in the attention task. Their idea was that, since working memory is a multicomponent system containing at least two separate stores for verbal and visuospatial information (Baddeley & Hitch, 1974), the interaction of specific types of load, target and distractor might decrease distraction, which was what they indeed observed (Kim et al., 2005). In addition, Dittrich and Stahl (2012) found that auditory selective attention is impaired only when both targets and distractors are verbal stimuli. These and similar findings (Park, Kim, & Chun, 2007) suggest that studies using different combinations of target and distractor stimuli can have different effects on performance.

### Neurocognitive task-engagement/distraction trade-off model

A more recent model (TEDTOFF, Sörqvist & Rönnberg, 2014) attempts to explain how and why working memory load can reduce distractibility, or act as ‘shield against distraction’ (Sörqvist & Marsh, 2015). According to the TEDTOFF model, two important aspects that affect concentration on the target task and neural suppression of distractors are variations in task difficulty and working memory capacity (WMC). Task difficulty may impact the ‘state’ (the more challenging the task the higher the concentration) and individual differences in WMC are considered the ‘trait’ of a given participant (Ilkowska & Engle, 2010).

One limitation of this model is that it is based exclusively on cross-modal distraction (mainly with a visual target and an auditory distractor), with evidence in its favor coming from intermodal task-based studies (Kim et al., 2005; Halin et al., 2015; SanMiguel et al., 2008; Sörqvist, Stenfelt, & Rönnberg, 2012; Sörqvist, Nöstl, A., & Halin, 2012; Sörqvist, Dahlström, Karlsson, & Rönnberg, 2016). Furthermore, as noted earlier, task relatedness of distractors may also modulate their effect (Kim et al., 2005; Park et al., 2007). Studies supporting the TEDTOFF model mainly used target and distractor tasks that were quite different in nature, for instance, letters in the WM task and tone sequences as distracting material (Sörqvist et al., 2016). To adjudicate between the TEDTOFF model and load theory, it is necessary to test whether there are settings in which concentration is enhanced in high load conditions even when targets and distractors share the same features.

### Single- versus dual-task paradigms: potential explanation of diverging results

One explanation why load theory and the TEDTOFF model predict such contrasting effects may lie in the paradigms employed in the studies that support each of these views, namely whether participants have to complete one or two tasks simultaneously. The growing body of research in favor of load theory prevalently comes from studies conducted within the so-called dual-task paradigm (Dalton et al., 2009; Kelley & Lavie, 2009, 2010; Lavie & De Fockert, 2005). A typical dual-task paradigm consists of a visuospatial task that tests inhibition of distractors to be performed in parallel with a working memory task with varying difficulty. Such settings, apart from the load on working memory and inhibition, potentially place high demand on shifting as the participant has to shift between the working memory set and the inhibition task (Diamond, 2013; Miyake & Friedman, 2012). Instead, reduced distraction has been found in experiments with a single task that contains a manipulation of working memory load and some distraction (Berti & Schröger, 2003; Guerreiro, Murphy, & Van Gerven, 2013; Güldenpenning, Kunde, & Weigelt, 2020; SanMiguel et al., 2008; Sörqvist, Stenfelt et al., 2012; Sörqvist, Nöstl et al., 2012) such that there is no need to divide and/or shift attention between two tasks. Constant shifting of attention between two unrelated tasks might leave little cognitive control for filtering out distraction and lead to poor focus on target stimuli. Augmenting the working memory load of a selective attention task itself could, on the other hand, increase engagement and leave little chance for processing distractors leading to phenomena like inattentional blindness or deafness (Macdonald & Lavie, 2011; Simons, 2000).

As regards the auditory domain specifically, only few experiments have been conducted so far but the existing findings are similar to those in the visual domain. Within the single-task paradigm, it has been shown that working memory load can result in reduced interference when both target and distractor are auditory stimuli (Berti & Schröger, 2003; Guerreiro et al., 2013), and the opposite has been found in dual-task settings (Dalton et al., 2009; Dittrich & Stahl, 2012). However, note that the former studies (Berti & Schröger, 2003; Guerreiro et al., 2013) were conducted using a dichotic listening paradigm. This calls into question the generalizability of their findings to less constrained settings.

In addition, the relationship between cognitive load and distraction may follow an inverted U-shaped curve (Simon, Tusch, Holcomb, & Daffner, 2016). This could imply that there is an optimal level of cognitive demand at which an individual can efficiently focus on the focal task and perform at their best. No further benefits of such cognitive load are expected to be observed if the task becomes too challenging, for the participant could become overloaded.

### The role of working memory capacity in distractibility

High WMC has often been associated with various attentional control mechanisms, predicting, for example, better performance in the flanker task (Heitz & Engle, 2007) or in the Stroop task (McCabe, Robertson, & Smith, 2005). Higher WMC reflects a more pronounced ability to control attention, not necessarily a larger memory store. Simple span tasks (like digit span) usually measure the storage component and complex span tasks (like the operation span task and *n*-back tasks) measure both storage and processing (Scharinger, Soutschek, Schubert, & Gerjets, 2017).

Although examined in only a limited number of experiments, WMC has been associated with the degree of interference caused by irrelevant auditory speech stimuli (Beaman, 2004; Sörqvist & Rönnberg, 2012; Zekveld et al., 2011). An interesting example of the direct effect of WMC on attentional capture by irrelevant but salient speech stimuli when attending to a target semantic message is presented in a study by Conway, Cowan, and Bunting (2001). Using a dichotic listening paradigm, they found that participants with higher WMC were less likely to notice a salient distractor.

Exploration of WMC effects on performance may also be useful to reveal a possible inverted U-shaped curve relationship existing, at group level, between task difficulty and distraction (Simon et al., 2016). If such a trend exists, we could expect that the decline in performance from a peak would occur earlier for individuals with low WMC. In a highly exploratory setting, we decided to collect two measures of WMC to test whether any interaction would be evident even with a relatively small number of participants. Operation span was assessed due to its conceptual similarity with *n*-back compared to other WMC tasks (Scharinger et al., 2017), and the auditory digit span was measured to explore whether a specific ability in remembering auditory information could be more related to auditory distractibility under load.

### The present study

To test whether auditory distraction can decrease under cognitive load, we adapted a single-task paradigm that combined a flanker task with the *n*-back task (a typical updating task, see Miyake & Friedman, 2012) where both target and distractor stimuli are letters (Scharinger et al., 2015). Such a design enabled us also to investigate whether distractors that are highly similar to targets would indeed create more interference under high load, as previously suggested (Kim et al., 2005; Park et al., 2007; Dittrich & Stahl, 2012). Furthermore, it would expand on the predictions of the TEDTOFF model to situations where both target and distracting information is presented in a unimodal task in the auditory domain. The advantages of using the *n*-back task are that working memory load can be easily adjusted and that its combination with the flanker task allows to study the interplay between task difficulty (or updating) and inhibition. Studies comparing performance in letter *n*-back tasks have found no differences between visual and auditory modalities (Rodriguez-Jimenez et al., 2009; Schumacher et al., 1996), although Schumacher et al. (1996) reported slower reaction times (RTs) in the auditory *n*-back task. Furthermore, previous research also indicates the feasibility of an auditory flanker task, showing the presence of a significant auditory conflict on incongruent trials, i.e., when flankers are represented by stimuli different from the central target stimulus (Chan, Merrifield, & Spence, 2005; Huang, Rossi, Hämäläinen, & Ahveninen, 2014).

In our task, distraction was represented by the congruency effect in the flanker task. The congruency effect in a flanker task is expressed in longer RTs on incongruent trials compared to congruent trials (Eriksen & Eriksen, 1974). We expected to find a two-way interaction between load and congruency such that distraction would be reduced in the 2-back condition compared to 0- and 1-back conditions but not continue to decrease in the 3-back condition, based on the idea of a possible inverted U-shaped function of this interaction (Simon et al., 2016). Reduction of the congruency effect was hypothesized to be no longer present in the 3-back condition as WMC could play a moderating role in the two-way interaction between load and congruency, given previous findings on the role of WMC in distractor processing (Conway et al., 2001; Kim, Wittenberg, & Nam, 2017). We hypothesized that participants with higher WMC would be less susceptible to flanker interference under high working memory load and could still benefit from working memory load in the 3-back condition. Performance of participants with lower WMC in the 3-back condition was expected to be more similar to 0- and 1-back conditions. This hypothesis is based on the idea of an optimal level of load that could be affected by individual differences in WMC (Sörqvist & Rönnberg, 2014).

Higher WMC was expected to be associated with an overall smaller congruency effect and higher accuracy. We expected performance in the operation span task to be a better predictor of individual differences in working memory capacity than the auditory digit span task (Scharinger et al., 2017). However, it should be noted that all hypotheses regarding the role of WMC, although preregistered, were exploratory and did not reflect the primary aim of the study.

The present study is based on a preregistered design and analysis (for more information, see https://osf.io/uh9dq/) and any deviations from this plan are noted and explained. Importantly, we employed a relatively novel modeling approach in statistical analysis, namely Bayesian generalized mixed-effects models were used (McElreath, 2016). This approach has several advantages, including the possibility to describe a phenomenon in probabilistic terms (i.e., with reference to the posterior probability distributions of parameters of interest), the possibility to introduce and test prior distributions, which formalize beliefs or previous evidence, and easier convergence for complex models (e.g., McElreath, 2016).

## Method

### Participants

Fifty adult volunteers (30 females) took part in this experiment. The mean age of participants was 22.62 years (SD 2.55, range 18–29) and 40 of them were undergraduate or postgraduate psychology students. The inclusion criteria were being a native Italian speaker, not having any history of neurological disease and being between 18 and 40 years of age. Two other participants also took part in the experiment, but were excluded due to technical issues in data collection. The study was approved by the local Ethics Committee and all participants signed an informed consent prior to the experiment.

The sample size was determined following the sequential Bayes factor design proposed by Schönbrodt, Wagenmakers, Zehetleitner, and Perugini (2017) (see also Schönbrodt & Wagenmakers, 2018). Evidence is quantified using the Bayes factor (BF). Full information on the rationale behind the determination of the sample size can be found in the preregistration document (https://osf.io/uh9dq/).

### Stimuli

**Fig. 1:**
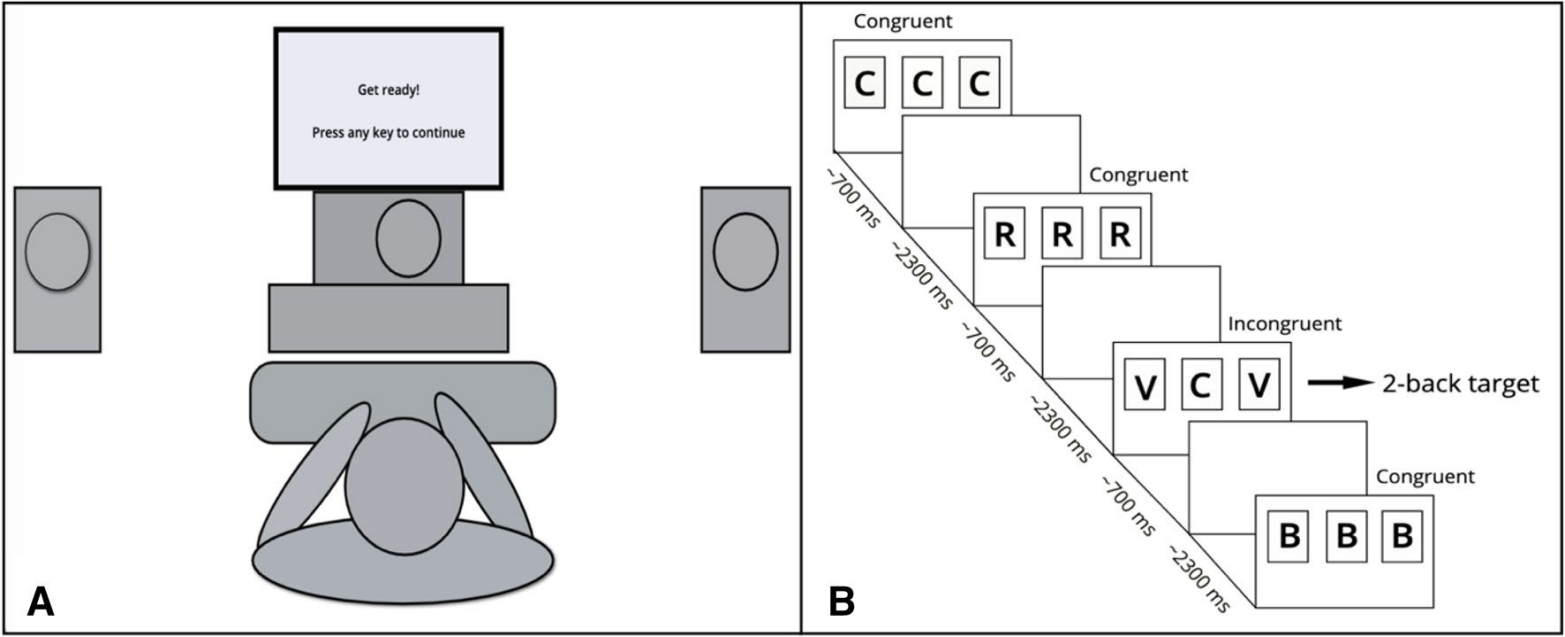
Experimental setup and task flow. **a**: Setup. The participant sat aligned with the central loudspeaker and used the keyboard to respond. The distance between the loudspeakers was 45 cm. The participant was instructed to attend to the central loudspeaker that was used to present the target stimulus and ignore the sounds coming from the side loudspeakers, i.e., flankers. **b**: Task flow. The stimuli were presented from three speakers as shown in **a**. Stimulus presentation lasted ~ 700 ms and the inter-stimulus interval was ~ 2300 ms. The image (not in scale) provides an example of a 2-back target, since the central letter (C) of the highlighted trial (arrow) matches the central letter presented two trials back

Stimuli consisted of a pair of letters presented via three loudspeakers arranged as shown in Fig. 1. Recordings of eight spoken letters (S, H, T, Q, B, V, R, C) were used to create the stimuli. One letter was presented by a male voice from the central loudspeaker, whereas the other letter was presented by a female voice from the two flanker loudspeakers located at a distance of 45 cm from the central one (see Fig. 1). On congruent trials, the letter presented from the central loudspeaker was flanked by the same letter, whereas on incongruent trials, it was flanked by a different letter. Letter pairs lasted approximately 700 ms.

Letters were recorded monophonically (44,100 Hz sampling rate, 16 bits resolution) in a single-walled IAC soundproof booth with a Shure SM58 microphone and subsequently edited using the Cool Edit Pro (Syntrillium Software Corp., Phoenix, AZ, USA) sound editing program. Two Italian speakers (one male and one female) were used to record the letters. Two voices were used to increase the discriminability between the letter played by the central loudspeaker and that played by the flanker loudspeakers. Single letter recordings were trimmed, matched in intensity, and combined in pairs. The onset of the letters in the pairs was adjusted so that the letters were perceived to start simultaneously. The male voice was always delivered to the central loudspeaker. The female voice was delivered to the flanking loudspeakers. To further facilitate the discrimination between the letter played by the central and the flanking loudspeakers, the central loudspeaker presented a letter 5 dB louder than the flanking letters: the sound pressure at the level of participants’ ears was set at 75 dBA for the central loudspeaker and 70 dBA for flanking loudspeakers.

### Procedure

Each participant completed two WMC tasks, followed by the experimental task (requiring selective attention). The first WMC task was the auditory digit span task (Cowan, Fristoe, Elliott, Brunner, & Saults, 2006). This task required participants to memorize auditory digit sequences and to reproduce them in the same order using the keyboard. Eight sequence lengths (from 2 digits to 9 digits) were included, with each sequence length consisting of two trials. The task started with the shortest sequence length. The participant progressed to longer sequences upon correct recall of at least one trial in a given sequence length. If both trials were incorrect, the task was discontinued.

Next, participants completed the automated shortened version of the operation span task (Ospan, Foster et al., 2015). A trial consisted of a math problem and letter recall. First, a math problem was presented (e.g., (3 * 2) − 4 = ?) and participants had to decide whether the solution that appeared after the problem (e.g., 3) was true or false by clicking on the corresponding word (‘vero’ for true or ‘falso’ for false). Each math problem was followed by a letter to be remembered. After each set of math problems and letters, a 4 by 3 matrix of letters (F, H, B, K, L, N, P, Q, R, S, T, and C) was presented, and participants were required to recall the letters in the order they had been presented. Five set sizes were used for the task, ranging from 3 to 7 and trials consisted of three sets of set size, totalling 75 trials.

Upon completion of the digit span and Ospan tasks, participants performed a selective attention task. The task consisted of an auditory hybrid *n*-back + flanker task that was adapted from Scharinger et al. (2015). On each trial, the participants were asked to attend to the letter played by the central loudspeaker and ignore the letter presented by the flanking loudspeakers (see Fig. 1). A blue LED on the central loudspeaker served as a fixation point. Participants heard sequences of letters and indicated via key press (L and D for target and non-target, counterbalanced between participants) whether the central letter of the current trial was identical to the central letter they heard *n*-trials back. The *n*-back matches are usually called *targets* and mismatches are called *non*-*targets* (Scharinger et al., 2015). Participants were instructed to respond as quickly and accurately as possible within 3000 ms from the stimulus onset (700 ms for the stimulus duration + 2300 ms of the inter-stimulus interval).

Four *n*-back load levels were used (0-back, 1-back, 2-back, 3-back), each level constituting a separate block of trials. Each block consisted of 120 trials, half of which were *n*-back targets. Two-thirds were congruent (i.e., central loudspeaker and flanking loudspeakers presenting the same letter), whereas one-third were incongruent (i.e., central loudspeaker and flanking loudspeakers presenting a different letter). This proportion was selected based on the study by Scharinger et al. (2015) to ensure that flankers were sufficiently distracting, since prior research has demonstrated that attention is more focused when there is a higher proportion of incongruent trials (e.g., Gratton et al., 1992; Logan and Zbrodoff, 1982; White et al., 2011). Moreover, incongruent trials were never followed by incongruent trials to avoid conflict adaptation, the so-called Gratton effect (Gratton et al., 1992). The first four trials were always congruent non-targets and were removed from any analysis. In the 0-back condition, participants were instructed that a certain letter (randomly selected among S, H, T, Q, B, V, G, or C) was the target and that on each trial they had to respond whether the letter presented by the central loudspeaker was the target or not by pressing the appropriate response key. As for the 1-, 2-, and 3-back conditions, participants had to respond whether the currently presented letter was the same as the letter heard one, two, or three trials back, respectively. The order of the four experimental *n*-back blocks was randomized for each participant and breaks were allowed between the blocks. Before the experimental trials, all participants completed training blocks of 20 trials for each *n*-back level. The threshold for proceeding to the next level was 60% accuracy. During training, participants’ accuracy was displayed at the end of a block for feedback. No feedback was given for experimental blocks. Overall, this task lasted approximately 30 min.

### Statistical analysis

In line with the preregistration (https://osf.io/uh9dq/), the analysis was divided into confirmatory (i.e., to assess a possible two-way interaction between load and congruency) and exploratory (i.e., investigating the relationship between WMC and task performance) sets of analyses.

All data analysis was conducted using the R software, version 3.6.2 (R Core Team, 2019). Data analysis was performed within a fully Bayesian framework. The *brms* package (Bürkner, 2017) was used for all purposes of data modeling. In the preregistration document, it was specified that both “BayesFactor” and “brms” packages would be used for model fitting depending on the variable at hand. However, we opted only for the latter for several reasons: (1) it allows to better handle the complex structure of our data, which requires modeling random effects; (2) it allows to fit generalized mixed-effects linear models, such as logistic regression (for accuracy), and gamma regression (for response times); (3) it allows to set and test subjective/informed priors; (4) it fits Bayesian models efficiently using the Markov chain Monte Carlo (MCMC) algorithm implemented in the STAN programming language.

The WAIC (Widely Applicable Information Criterion, lower is better; Watanabe, 2010) fit index was used for all purposes of model comparison and statistical inference. From it, an evidence ratio (ER) was calculated (cf. Burnham, Anderson, & Huyvaert, 2011; also named “relative likelihood”, Wagenmakers & Farrell, 2004). The ER quantifies the evidence in favor of one model being better than an alternative model. When comparing two models with vs without a given effect of interest, the ER quantifies evidence in favor or against the effect of interest (i.e., the relative likelihood of H_1_ vs H_0_).

This represents a minor deviation from the preregistration, where using the Bayes factor (BF) was assumed. ER was preferred over BF due to the complexity of the mixed-effects models with three-way interactions that made it difficult to reliably compute the BF, leading to several warnings of the “Bayes_factor” function (“brms” package). In contrast, the WAIC index is more robust. BF and ER were highly similar, but BF exaggerated the evidence as compared to the ER for some comparisons.

ERs were interpreted using a threshold of 4 (or 1/4) for moderate evidence. In addition, the posterior distributions of model parameters were examined to gain insight into the specific effects of interest. Model parameters were considered as “non-null” when the 95% highest posterior density intervals (HPDI) excluded zero. HPDI is a Bayesian analog of the frequentist confidence intervals.

### Confirmatory analysis

Our primary hypothesis regarded the two-way interaction between *n*-back level and the congruency effect. Both factors are within-participants: *n*-back Load (0-back, 1-back, 2-back, and 3-back; 2-back was the baseline level) and Congruency (flanker stimuli: congruent vs. incongruent flankers; congruent was the baseline level). As regards Load, 2-back was set as the baseline because we expected reduced a congruency effect in the 2-back condition compared to 0- and 1-back conditions, but no further increments in this facilitation mechanism in the 3-back condition. Setting 2-back as the baseline for the Load factor was the most direct way to formalize our expectations. The dependent variable was RTs of correct trials. Accuracy was also examined in a separate analysis.

As data consisted of a series of repeated responses by participants, the analysis was performed by fitting Bayesian generalized linear mixed-effects models (GLMM). Congruency, Load, and their interaction were entered as the fixed effects of interest. Participants were entered as random effects in all models. We fitted a series of models with random intercepts by participants. As Congruency and Load are both factors, they were dummy coded in the model. The formula of the main model of interest is presented in the Supplemental Materials, Section 2. Convergence of the parameter estimates was assessed with the ‘Rhat’ (potential scale reduction factor on split chains). Rhat was below 1.01 for all parameters, indicating good convergence (at convergence, Rhat = 1.00).

RTs were modeled with the gamma distribution with the “Log” link function (see Supplemental Material, Section 2). This assumes that RTs vary on a logarithmic scale, with variance increasing with longer RTs. This reflects the actual distribution of the data (see descriptive statistics in Table 1 where higher standard deviations (SDs) correspond to higher means for the RT variable), and is generally the case for RTs.

**Table 1:** Descriptive statistics for RTs and accuracy by Load and Congruency (calculated before data filtering)

Load	Congruent		Incongruent	
	Mean	SD	Mean	SD
Response time (ms)				
0-Back	752	151	802	157
1-Back	834	211	889	242
2-Back	1085	262	1113	289
3-Back	1289	286	1306	306
Accuracy (proportion)				
0-Back	0.98	0.03	0.98	0.03
1-Back	0.95	0.07	0.96	0.08
2-Back	0.89	0.09	0.87	0.10
3-Back	0.79	0.09	0.77	0.10

However, the use of the gamma distribution represents a potential deviation from the preregistration document where the assumption concerning the distribution of RT data was not discussed, but could be interpreted as tacitly suggesting the normal (Gaussian) distribution. Therefore, to ensure that the interpretation of the results was not bound to the assumptions on gamma/Gaussian distribution of RTs, we subsequently repeated all analyses using the classical linear mixed-effects models (LMM, with the identity link function). This additional analysis is reported in the Supplemental Materials, Section 4. Finally, accuracy was modeled using a logistic regression, due to the inherently binomial (i.e., series of correct/incorrect responses) nature of this dependent variable.

### Definition of prior knowledge

In the preregistration, no use of informed priors was mentioned. Therefore, a first series of models were fitted and analyzed using uninformed default priors for all parameters. However, given the adoption of a fully Bayesian approach, we subsequently decided to formalize a set of informed priors based on the meta-analysis of the relevant previous literature. Prior formalization concerned the model parameters of the interaction of interest. This was limited to RTs, as it is the dependent variable for which a Load by Congruency interaction was expected and theoretically motivated. For a full description of this procedure, refer to the Supplemental Materials, Section 1.

## Results

### Descriptive statistics and filtering data

Descriptive statistics for RTs and accuracy, divided by Load and Congruency, are reported in Table 1. These were calculated on the raw data, after filtering data for subsequent analysis.

According to the preregistration document, all blocks with accuracy below 60% were excluded. No participant was below this threshold in the overall performance across the four experimental blocks (ranging from 62.9 to 97.4%). Nonetheless, 13 participants had an accuracy below 60% in one or more blocks, for a total of 15 blocks (representing 7.5% of all 200 blocks). The use of mixed-effects linear models allowed to remove only these blocks from the analysis. In addition, RTs below or above 3 SD of the participant’s own mean calculated in each condition (i.e., block) were removed. These anticipated/delayed responses constituted only 1.1% of the total observations.

### Confirmatory analysis: RTs

**Fig. 2:**
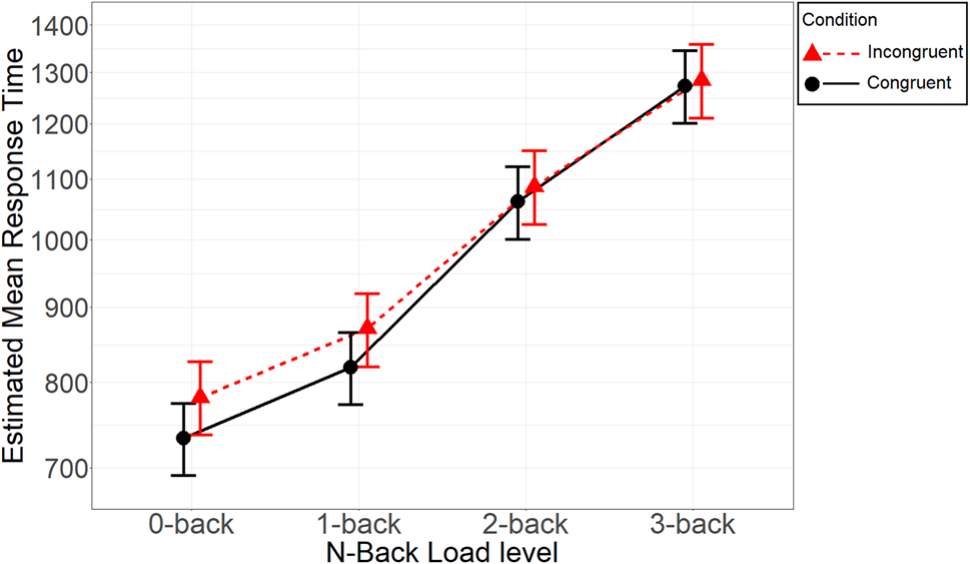
Estimated mean response time as a function of Load level. *Note* Error bars represent 95% Bayesian credible intervals of the posterior estimates calculated with the percentile method. *Y* axis scale was log-transformed to reflect the use of gamma distribution

Uninformed default priors were used for this first confirmatory analysis. The 4 × 2 interaction between Load and Congruency was supported by strong evidence, ΔWAIC = 16.4, ER > 1000. In addition, both main effects of Load and Congruency were also supported by strong evidence; in both cases, ER > 1000 (ΔWAICs were 6339.9 and 74.5 respectively). The estimated average RTs by Load and Congruency are shown, along with their credible intervals, in Fig. 2. Full details of the parameters of the model can be found in Table 2. As shown in Fig. 2, the congruency effect (i.e., difference in average RT between congruent and incongruent condition) was more pronounced at the 0-back and 1-back Load level than at either 2-back or 3-back Load level.

**Table 2:** Details on the posterior distributions of model parameters

Response variable/model coefficient	Estimate	SE	95% HPDI
Response time (GLMM with gamma family)			
*β*_0_ (intercept)	6.97	0.03	(6.90, 7.02)
*β*_1_—Load: 0-back	− 0.37	0.01	(− 0.39, − 0.36)
*β*_2_—Load: 1-back	− 0.26	0.01	(− 0.27, − 0.24)
*β*_3_—Load: 3-back	0.18	0.01	(0.16, 0.20)
*β*_4_—Congruency: Incongruent	0.02	0.01	(0.00, 0.04)
*β*_5_—Load × Congruency: 0-back	0.04	0.01	(0.02, 0.07)
*β*_6_—Load × Congruency: 1-back	0.04	0.01	(0.01, 0.06)
*β*_7_—Load × Congruency: 3-back	− 0.01	0.01	(− 0.04, 0.01)
Shape^a^	9.82	0.10	(9.63, 10.02)
Accuracy (GLMM with binomial family)			
*β*_0_ (intercept)	2.27	0.11	(2.04, 2.50)
*β*_1_—Load: 0-back	2.50	0.17	(2.15, 2.82)
*β*_2_—Load: 1-back	1.00	0.10	(0.81, 1.20)
*β*_3_—Load: 3-back	− 0.90	0.08	(− 1.05, − 0.76)
*β*_4_—Congruency: incongruent	− 0.07	0.10	(− 0.26, 0.12)
*β*_5_—Load × Congruency: 0-back	− 0.24	0.26	(− 0.78, 0.26)
*β*_6_—Load × Congruency: 1-back	0.32	0.18	(− 0.02, 0.68)
*β*_7_—Load × Congruency: 3-back	0.01	0.12	(− 0.24, 0.25)

These models were fitted by using uninformed default priors. “Estimate” represents the mean value of the posterior distribution; SE (standard error of the estimate) represents its SD. Baseline levels were: “2-back” for Load; “congruent” for Congruency

*HPDI* highest posterior density interval, *GLMM* generalized linear mixed-effects model

^a^Shape refers to the estimated “shape” parameter of the gamma distribution. More details about the model mathematical formula can be found in the Supplemental Material, Section 2

The interaction parameters indicate the difference in congruency effect at the alternative levels (0-, 1-, and 3-back) in comparison to the baseline level (2-back). They can be roughly interpreted as the estimated difference in the congruency effect between the alternative level and the baseline as a proportion of the baseline value. Translated into predicted values, Fig. 2 shows that the congruency effect at 0-back and 1-back was about 30–40 ms larger than at 2-back, whereas the congruency effect at 3-back was virtually the same as at 2-back. The estimates were as follows: for 0-back, *B* = 0.04, 95% HPDI (0.02, 0.07); for 1-back, *B* = 0.04, 95% HPDI (0.01, 0.06); for 3-back, *B* = − 0.01, 95% HPDI (− 0.04, 0.01). Therefore, there was substantial evidence that the congruency effect in 0- and 1-back conditions (virtually identical) differed from 2-back, while there was no evidence for the 3-back contrast.

Finally, congruency effects were also calculated at the individual level, examining the random effects of the mixed-effects models. Broadly speaking, it can be concluded that the pattern represented by the averaged effect was also present in most participants; further information on the individual variability of the effect can be found in Supplemental Materials, Section 3.

### Prior knowledge and its impact on estimated parameters for RTs

As specified in the Statistical analysis section, a set of informed priors, based on five previous studies conducted within the single-task paradigm similar to the present experiment (Berti & Schröger, 2003; Guerreiro et al., 2013; Güldenpenning et al., 2020; SanMiguel et al., 2008; Scharinger et al., 2015), was formalized for the interaction parameters of the model and used in a newly fitted model. All details are reported in Supplemental Materials, Section 1.

**Fig. 3:**
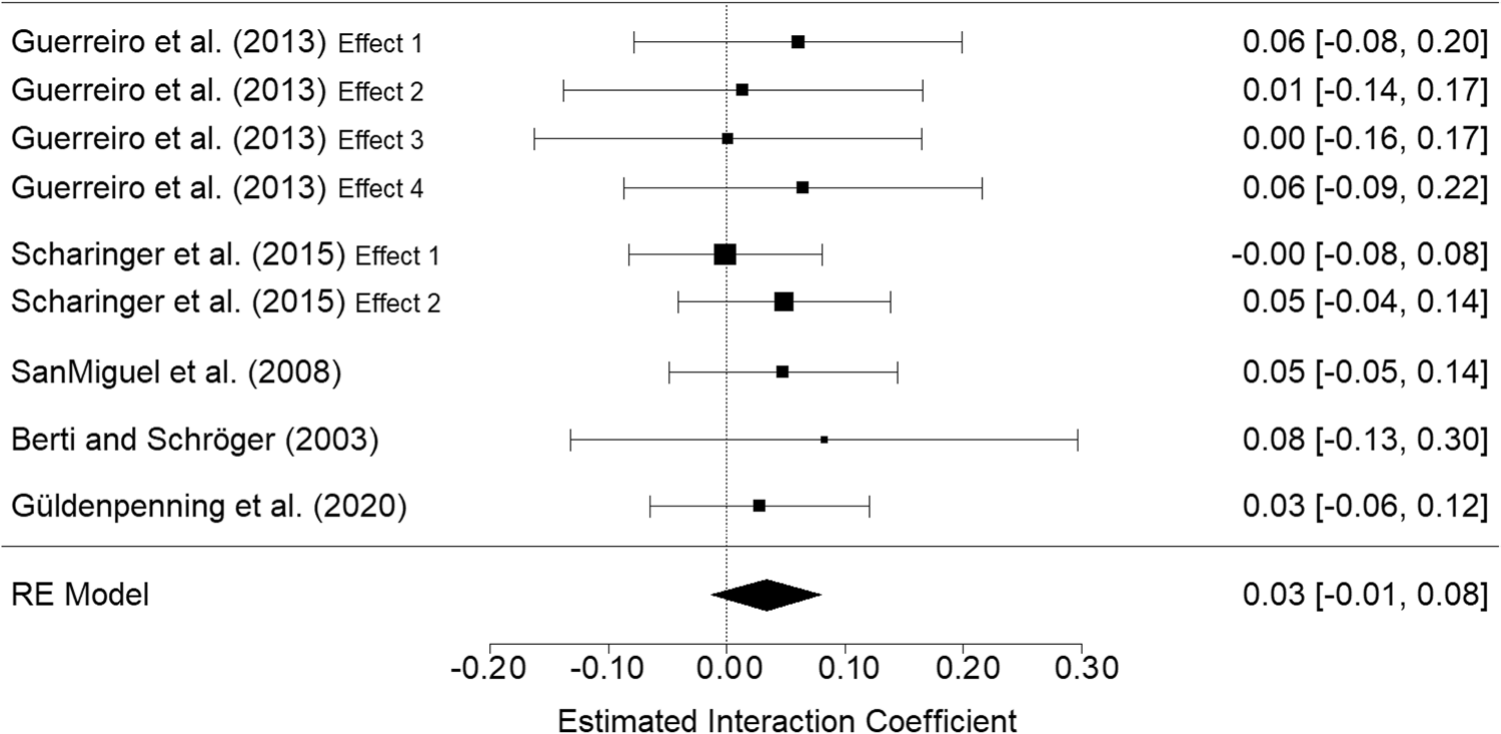
Forest plot and overall meta-analytic estimate for the interaction parameter (gamma family). *Note* The effects refer to the interaction parameter of interest, which is the estimated difference in the Congruency effect in a “low” vs. a “high” Load conditions. Positive values indicate larger congruency effects in “low” than in “high” Load conditions. (For interpretation of the metrics, see the text or find more easily interpretable linear parameters in Figure S6 in Supplemental Materials.) Error bars represent 95% CIs

The forest plot in Fig. 3 shows the estimated interaction parameters of interest for the effects reported in these previous studies, as well as the meta-analytic estimate. For interpretation, note that these effects indicate the estimated difference in the congruency effect observed in the “low” in comparison to the “high” Load level. They are positive since at low load the congruency effect tends to be larger than at high load. As we set the “high” Load condition (2-back) as the baseline in our analysis, we considered the alternative levels 0- and 1-back as “low” Load levels. For the parameter concerning the 3-back level, which represents the highest Load condition, the sign of the prior parameter had to be inverted. The overall meta-analytic parameter was 0.033, and it can be roughly interpreted as follows: the difference in congruency effect between the “high” and “low” load corresponds to 3.3% of the baseline. For example, if the estimated average response time at “high” load in the congruent condition (i.e., at baseline) is 1000 ms, then the estimated difference in congruency effect between “high” and “low” Load would be approximately 33 ms.

**Fig. 4:**
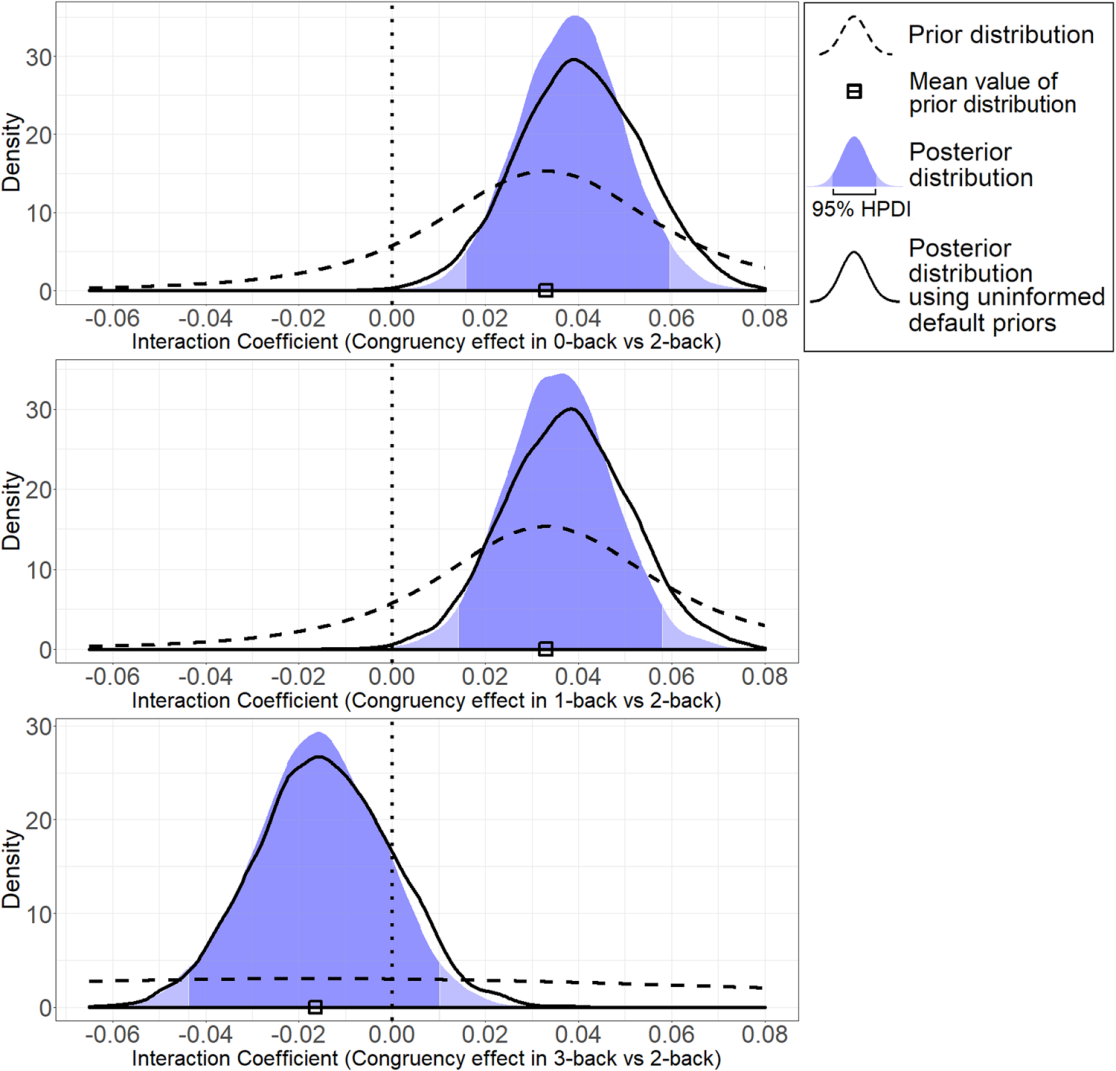
Prior and posterior distributions for the three interaction parameters on response times

As we set the “high” Load condition (2-back) as the baseline in our analysis, and we considered the alternative levels 0- and 1-back as “low” load levels, we directly used the meta-analytic estimate of 0.033 as the mean values of the (Normal) prior distributions for these two parameters (note that a regression parameter for a categorical factor represents the comparison between the alternative level [i.e., low load] and the baseline level [i.e., high load]). The SDs of these parameters were set at 0.024, which corresponds to the SE of the meta-analytic estimate. As detailed in the Supplemental Materials, Section 1, we assumed that 3-back could represent an even higher Load condition than 2-back. However, in the preregistration it was noted that we would expect no further increment in the congruency effect at the 3-back as compared to the 2-back level. Therefore, we set the mean prior value for the “3-back vs 2-back” parameter at only half the meta-analytic estimate, and we set large uncertainty on its distribution (i.e., five times the SD used for the other parameters, or SD = 0.12), which was practically equivalent to a non-informed prior distribution, as shown by the very flat prior distribution in the bottom panel of Fig. 4. Lastly, for the “3-back vs 2-back” parameter the comparison no longer indicated “low vs high Load” (as in the “0-/1-back vs 2-back” parameters), but “very high vs high Load”. Therefore, here we had to invert the sign of the meta-analytic estimate (i.e., we had to use a negative instead of positive sign).

Prior distributions of the three interaction parameters were set as Student’s t with 3 degrees of freedom, with mean and SD based on the meta-analysis as follows. For coefficients “Load × Congruency: 0-back” and “Load × Congruency: 1-back”, *M* = 0.033, SD 0.024: for coefficient “Load × Congruency: 3-back”, *M* = − 0.017, SD 0.120 (2-back is the baseline level).

The new model embedding the informed priors provided estimates that were nearly identical to the previous one. This was provided by the fact the informed priors were remarkably in line with the likelihood of our data. Figure 4 shows the prior and posterior distributions for the interaction parameters of the GLMM.

### Confirmatory analysis: accuracy

**Fig. 5:**
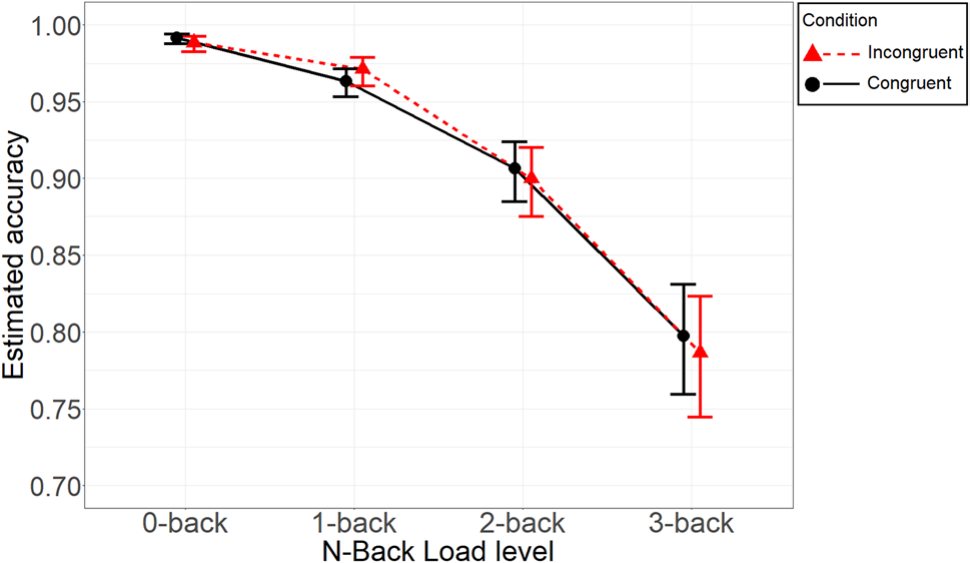
Estimated probability of correct response as a function of Load and its interaction with Congruency. *Note* Error bars represent 95% Bayesian credible intervals of the posterior estimates calculated with the percentile method

Evidence suggested a lack of interaction between Load and Congruency on accuracy, ΔWAIC = − 1.0, ER = 0.61 (note that it failed to reach the threshold of 1/4 we set for moderate evidence in favor of H_0_). There was similar evidence also against a main effect of Congruency ΔWAIC = − 1.5, ER = 0.47. Finally, however, there was strong evidence in favor of a main effect of Load, ΔWAIC = 1484.4, ER > 1000 (accuracy decreased with increasing load). The estimated probabilities of correct response by Load and Congruency are shown, along with their credible intervals, in Fig. 5. Full details on model parameters can be found in Table 2.

### Exploratory analysis on the role of WMC

In a follow-up analysis, we also examined the role of WMC as a potential moderator of the Load by Congruency interaction, to assess how this two-way interaction alters after adjusting for the continuous covariates of the Ospan score and auditory Digit span. Consideration of models’ contrasts is conditional to finding relevant interactions for their respective effects. As stated in the preregistration, along with the rationale for performing it, this analysis has to be considered exploratory as we did not expect to have sufficient statistical power for assessing this possible interaction.

There was modest evidence against a main effect of digit span on accuracy, ΔWAIC = − 0.5, ER = 0.78. As for Ospan partial, there was modest evidence in favor of its main effect on accuracy, ΔWAIC = 1.3, ER = 1.92, which was moderated by Load, ΔWAIC = 23.9, ER > 1000. Model parameters suggested that Ospan partial had a positive association with accuracy at lag 0, *B* = 0.41, 95% HPDI (0.12, 0.69), a null association at lag 1, *B* = − 0.03, 95% HPDI (− 0.27, 0.23), again a positive association at lag 2, *B* = 0.36, 95% HPDI (0.14, 0.57), and a null association at lag 3, *B* = 0.03, 95% HPDI (− 0.18, 0.26). Digit span did not interact with congruency, ΔWAIC = − 1.7, ER = 0.43, nor did Ospan partial, ΔWAIC = − 1.8, ER = 0.41. Moreover, the correlation between Ospan partial and Digit span was only *r* = 0.27, 95% CI (− 0.01, 0.51).

Given that, as expected, evidence for an interaction between Load and Congruency emerged for RTs but not for accuracy, we explored how such interaction could be moderated by WMC only for RTs. As regards the digit span, evidence was against a three-way interaction of interest, as it favored the model excluding it, ΔWAIC = − 4.4, ER = 0.11. There was no evidence for a main effect of Digit span either, as the model including it in addition to the Load × Congruency interaction had worse fit than the model including the interaction alone, ΔWAIC = − 0.3, ER = 0.86. As for the Ospan partial, due to the complexity of the model including the three-way interaction, neither WAIC nor BF could be computed reliably. Therefore, we re-computed the same models via maximum likelihood and quantified evidence using the AIC (Akaike information criterion; Akaike, 1974; this is analogous to the WAIC in the frequentist context), and the ER related to it (Burnham et al., 2011). There was evidence against the three-way interaction, ΔAIC = − 4.2, ER = 0.12. There was no evidence for a main effect of Ospan partial either, ΔWAIC = − 0.1, ER = 0.95.

## Discussion

The present study investigated the effect of working memory load on auditory selective attention within a single-task paradigm where the levels of load and interference were manipulated within the same task. It also explored how WMC can potentially change the interaction between working memory load and interference. To our knowledge, this is one of the few studies on selective attention under cognitive load that implemented a preregistered design and analysis plan. We found that interference from distractors diminished with higher verbal working memory load despite the similarity of targets and distractors and the fact that they were both presented in the auditory domain. These findings suggest that increasing the load on working memory can facilitate attentional focus even in the presence of salient distractors potentially due to a higher demand on the attentional resources involved in the working memory task.

Manipulations of *n*-back load and flanker interference were both successful. RTs substantially increased in more demanding load conditions, and participants were slower on incongruent trials as compared to congruent. Importantly, we found that the congruency effect interacted with load. Interference from auditory flankers progressively decreased with increasing working memory load, virtually disappearing in the 2-back condition. Therefore, the main hypothesis that attentional focus can benefit from high cognitive load was ultimately supported. The difference between these results and the studies that have found increased interference under higher cognitive load is another indicator of the need to consider the setting in which different cognitive functions are manipulated. As noted earlier, we assume that, in this study, interference decreased in higher load conditions since the participant had to focus on one task rather than shifting between two different tasks as in dual-task paradigms. These findings build on the previous research that showed that cognitive load can improve attentional performance even when distracting stimuli are presented in the same modality as the target information (Berti & Schröger, 2003; Guerreiro et al., 2013; Güldenpenning et al., 2020; Scharinger et al., 2015). They further expand on the TEDTOFF model (Sörqvist & Rönnberg, 2014) by showing that this interaction is observed also when both target and distractor are auditory stimuli.

Our findings are novel in that they show that increased cognitive load can enhance focus even when the same auditory speech stimuli are used for both target and distractor stimuli. They contradict prior findings suggesting that distractibility selectively increases only when both distractors share the same features with the working memory task, i.e., both are verbal stimuli (Dittrich & Stahl, 2012; Kim et al., 2005; Park et al., 2007). However, it should also be noted that those experiments were conducted using the dual-task paradigm, hence, although their results cannot be directly compared with the present study, the fact that they find such contrasting effects based on different working memory load, target, and distractor manipulations calls for more research to test the validity of the used paradigms. Indeed, Gil-Goméz De Liaño, Umiltà, Stablum, Tebaldi, & Cantagallo (2010) failed to replicate the finding on the effect of relatedness of distractors to the working memory task (Kim et al., 2005). That said, we cannot exclude that such a relationship can emerge in different contexts. Given that we only used letters as stimuli, our results actually support the view that interference is reduced in higher load conditions when the WM task and distractors overlap. However, our experiment did not include a separate attention task with targets presented in another modality. It could be argued that as the same stimuli are used as targets and distractors in the flanker task, distractor stimuli cannot be considered totally irrelevant, which is different from studies that use as distractors, for example, some environmental sounds that are clearly unrelated to the task (Escera, Alho, Winkler, & Näätänen, 1998; SanMiguel et al., 2008).

In line with our expectations, the congruency effect was not significantly different between the 2- and 3-back conditions. This could be interpreted as a preliminary and modest suggestion of an existence of the inverted U-shaped curve of the relationship between cognitive load and distractibility. However, it is worth considering that although significant, the level of distraction itself was not high in any of the load conditions, which is similar to prior studies in both the visual (Güldenpenning et al., 2020; Scharinger et al., 2015) and the auditory (Berti & Schröger, 2003; Guerreiro et al., 2013) domains. Besides, note that the male voice was always used as the target stimulus and the female voice was the distractor and one possibility is that distinguishing a male voice from a female distractor might not present a great difficulty. Additionally, 3-back was chosen as the highest load level in the present study given that previous studies that tested the same interaction mostly used 2-back as their highest working memory load (Guerreiro et al., 2013; Scharinger et al., 2015; see Murphy, Groeger, & Greene, 2016 for a review) and our expectations about 3-back condition were more speculative in this respect. To further explore the possibility of an inverted U-shaped trend, future studies can use more salient distractors and even more challenging working memory load conditions that would further deplete capacity-limited attentional control resources in the presence of potent distractors (Simon et al., 2016). Any eventual comparisons of WMC scores should consider between-group differences and adjust the working memory load accordingly, which would be helpful to improve the existing methods in education, workplace, and speech recognition training programs (Ballesteros et al., 2017).

Our findings are in accord with the previous studies using a single-task paradigm both in the auditory (Berti & Schröger, 2003; Guerreiro et al., 2013), visual (Guerreiro et al., 2013; Güldenpenning et al., 2020; Scharinger et al., 2015), and cross-modal (SanMiguel et al., 2008) experiments. Such domain generality of the effect is compatible with the presence of limited, and relatively unspecific, attentional resources whose availability plays a major role in determining the impact of task difficulty upon different processes (Bonato, Umiltà, & Zorzi, 2013). They are also consistent with the role of task difficulty (which can be also determined by factors other than working memory load) as an obvious yet neglected performance modulator (Lisi, Bonato, & Zorzi, 2015). Evidence derived from non-visual domains is crucial for testing the extent to which the effects found within the prevalently studied visuospatial attention domain are not modality-specific but rather reflect general characteristics of attentional functioning (Spence, 2020). Importantly, our findings suggest that this paradigm can be used to study processing of speech stimuli in the presence of background which is crucial for the populations with impaired speech recognition in noise.

As in the majority of previous studies (Murphy et al., 2016), we sought and found the interaction between task engagement and cognitive load in reaction time but not in accuracy. This indicates that participants can still meet task demands, but at the expense of speed. However, in our daily lives, we often face situations where some goal attainment requires immediate response, as for example, when we need to attend to and memorize the features of a given object that is only present for a limited time. One further direction, therefore, could be designing paradigms that impose greater demands on speed, by, for instance, using stimuli that disappear after a short time interval. Moreover, studies using neuroimaging techniques or other psychophysiological methods could provide some useful insights into the stage of processing which would be responsible for either a slower response or an error. It is important to account for these different effects as in some cases, changes in brain function can affect accuracy while leaving the speed of correct responses intact (Laures, 2005).

Neither of our WMC measures affected the two-way interaction between Load and Congruency. As mentioned earlier, this effect was difficult to account for given our sample size. Besides, the correlation between the two measures was quite low compared to what has previously been reported (Kane et al., 2004), and while Ospan weakly predicted accuracy in *n*-back, digit span did not. This could be either due to the homogeneity of the tested sample or its insufficiency. However, the fact that WMC did not predict even the congruency effect in the flanker task might provide some additional support to the studies reporting that performance in the flanker task is independent from WMC measured by complex span tasks (Friedman & Miyake, 2004; Wilhelm, Hildebrandt, & Oberauer, 2013). On the other hand, many studies have shown that WMC can predict performance in tasks that involve inhibition or shifting, for instance, but do not include an obvious memory component (see Kane et al., 2007, for a review). Wilhelm et al. (2013) explain these inconsistencies in findings by the ill-suited analytic approach adopted by those who find an interaction. Particularly, they suggest that studies that find WMC to be a predictor in, for example, the dichotic listening task (Conway et al., 2001), a go/no-go task (Redick & Engle, 2006), and Stroop task (Kane & Engle, 2003), all use extreme group comparisons which leads to an overestimation of effect sizes. Secondly, they suggest that along with WMC measured by complex span tasks, at least updating, inhibition, and shifting abilities should also be assessed as they could conflate variance if left latent (Wilhelm et al., 2013). Future studies with substantially larger samples than ours could focus on the role of WMC on distractibility under cognitive load by also including a comprehensive assessment of executive functions.

More ecologically valid paradigms that could better mimic real life settings or situations where a specific type of distraction is most detrimental to task engagement would be useful to inform real life applications of findings on selective attention. Such paradigms are crucial to account for the plethora of possible task combinations we are exposed to in our daily lives. Importantly, any experimental approach to the study of human attention that triggers interactions across domains might be closer to everyday life demands than it seems.

## Conclusion

This study suggests that cognitive load can reduce distraction from irrelevant auditory sources when people need to attend to a single source of auditory information in noisy environments. This finding has the potential to shed light on speech recognition issues that occur for various reasons, such as aging, hearing loss, or brain injury, manifested at different stages of development. Furthermore, it can also help to better understand the everyday circumstances that lead to higher distraction from auditory stimuli. This occurs, for example, when voice commands, cell phones, or other voice-activated devices are used while performing another activity requiring focused attention such as driving or walking or simply working in an office.

This effect was observed in a task that did not involve a particular demand on shifting and where only letter stimuli presented in the auditory domain were used. It implies that cognitive load can be used as an aid with which to enhance concentration when salient distractors of similar nature are present in the environment. However, as distractors used in the present study did not represent any potential threat or other type of highly relevant information regardless of the current task goals, we suggest that these findings be interpreted with caution. Neither Ospan nor auditory digit span were found to predict performance in the selective attention task which likely stems from the lack of participants to test this between-group difference, but only future experiments can answer this question. There is scope to explore what is an optimal level of cognitive load that facilitates attentional focus, which, in our case, was 2- and 3-back conditions. These results can be used both as an approximate estimate of task difficulty level and subsequently translated into cognitive demand tasks of similar difficulty and also expanded on by investigating the point at which the relationship between interference and cognitive load is reversed.

## Electronic supplementary material

Below is the link to the electronic supplementary material.

Supplementary material 1 (PDF 1061 kb)

## Data Availability

The data for this experiment are available from the OSF repository at the following https://osf.io/uh9dq/.
