# Supplementary material for: Auditory selective attention under working memory load
Source: Psychol Res. 2020 Nov 4;85(7):2667–81. doi: 10.1007/s00426-020-01437-7 (PMC8440250; doi:10.1007/s00426-020-01437-7)
Supplement: Supplementary file 1 — Supplementary material 1 (PDF 1061 kb) [file 426_2020_1437_MOESM1_ESM.pdf]

Psychological Research

Supplemental Materials for

Auditory Selective Attention Under Working Memory Load

Rena Bayramova, Enrico Toffalini, Mario Bonato, Massimo Grassi

Correspondence to:
renabayramova@outlook.com
enrico.toffalini@yahoo.it

October 6, 2020

Contents

1 Detailed Description of the Analysis Plan **1**
1.1 Confirmatory Analysis . . . 1
1.2 Definition of Prior Knowledge . . . 2
1.3 Exploratory Analysis . . . 3

2 2 Formalization of the (G)LMM on Response Times **4**
2.1 Linear model . . . 4
2.2 Link function . . . 4

3 Individual-level Congruency Effects (analysis of random effects) **5**

4 Results Obtained Using LMM (i.e., with normality assumption) on RTs **10**

5 Prior Knowledge Formalization for the LMM on RTs and its Impact on the Posterior Distributions **12**

References **15**

List of Figures

1 Individual congruency effect in 0-back (ms), ordered by decreasing estimated effect (top to bottom). *Note:* Error bars represent 95% Bayesian credible intervals (BCI) calculated with the percentile method. The black rhombus above the X-axis represents the estimated population-level effect with its 95% BCI. 6
2 Individual congruency effect in 1-back (ms), ordered by decreasing estimated effect (top to bottom).*Note:* Error bars represent 95% Bayesian credible intervals (BCI) calculated with the percentile method. The black rhombus above the X-axis represents the estimated population-level effect with its 95% BCI. 7
3 Individual congruency effect in 2-back (ms), ordered by decreasing estimated effect (top to bottom).*Note:* Error bars represent 95% Bayesian credible intervals (BCI) calculated with the percentile method. The black rhombus above the X-axis represents the estimated population-level effect with its 95% BCI. 8
4 Individual congruency effect in 3-back (ms), ordered by decreasing estimated effect (top to bottom).*Note:* Error bars represent 95% Bayesian credible intervals (BCI) calculated with the percentile method. The black rhombus above the X-axis represents the estimated population-level effect with its 95% BCI. 9
5 Estimated mean RT as a function of the Load by Congruency interaction, from the GLMM using the Gamma distribution for modelling RT. *Note:* Error bars represent 95% Bayesian credible intervals of the posterior estimates calculated with the percentile method. 11
6 Individual congruency effect in 0-back (ms), ordered by decreasing estimated effect (top to bottom). 13
7 Prior and posterior distributions for the three interaction parameters on RTs. . . . 14

List of Tables

1 Descriptive statistics 4
2 Details on the posterior distributions of model parameters using LMM. 12
3 Prior distributions formalized for the interaction parameters of interest. 13

1 Detailed Description of the Analysis Plan

The analysis was divided into confirmatory (i.e., to assess a possible two-way interaction between load and congruency) and exploratory (i.e., investigating the relationship between WMC capacity and task performance) sets of analyses. Here, we describe the details of the analyses along with explanations of some minor deviations from the preregistered analysis plan and the originally planned analysis.

All data analysis was conducted using the R software, version 3.6.2 (R Core Team, 2019). In line with the preregistration, a fully Bayesian framework was used for data analysis. This approach has several advantages, including the possibility to describe a phenomenon in probabilistic terms (i.e., with reference to the posterior probability distributions of parameters of interest), the possibility to introduce and test prior distributions, which formalize beliefs or previous evidence, and easier convergence for complex models (e.g., McElreath, 2016). The "brms" package (Bürkner et al., 2017) was used for all purposes of data modelling. In the preregistration document, it was specified that both "BayesFactor" and "brms" packages would be used for model fitting depending on the variable at hand. However, we eventually opted only for the latter for several reasons: 1) it allows to better handle the complex data structure of the data, which requires modelling of random effects (as the dependent variables consist of series of several responses by participant); 2) it allows to fit generalized mixed-effects linear models, such as logistic regression (for accuracy), and Gamma regression (for response times, RTs); 3) it allows to set and test subjective/informed priors (using a range of different distributions); 4) it fits Bayesian models efficiently using the Markov chain Monte Carlo (MCMC) algorithm implemented in the STAN programming language.

The WAIC (Widely Applicable Information Criterion, lower is better; Watanabe, 2010) fit index was used for all purposes of model comparison and statistical inference. From it, an evidence ratio (ER) was calculated (cf. Burnham et al., 2011; also named "relative likelihood", Wagenmakers & Farrell, 2004). The ER quantifies the evidence in favor of one model compared to an alternative model. When comparing two models with vs without a given effect of interest, the ER quantifies evidence in favor or against the effect of interest – i.e., the relative likelihood of H1 vs H0. This represents a minor deviation from the preregistration, where using the Bayes factor (BF) was assumed. ER was preferred over BF due to the complexity of the mixed-effects models with 3-way interactions that made it difficult to reliably compute the BF, leading to several warnings of the "bayes_factor" function ("brms" package). In contrast, the WAIC index is more stable. Nonetheless, we reported BF along with ER in an additional series of analyses described in detail in the present Supplemental materials, Section3. BF and ER were highly similar but BF exaggerated the evidence vs ER for some comparisons (which was more conservative).

In fact, the same logic can be applied when interpreting ER and BF in model comparison. BF > 3 is generally reported (e.g. Schönbrodt et al., 2017), as moderate/sufficient evidence in favor of H1, BF > 10 as strong evidence, BF > 100 as very strong evidence. The same criteria can be used to quantify evidence for H0 (with BF $< 1/3$, 1/10, 1/100, respectively). As indicated in the preregistration, we interpreted ER using the same thresholds for strong and very strong evidence but a slightly stricter threshold of 4 (or 1/4) for moderate evidence.

In addition, we examined the posterior distributions of model parameters to gain insight into the specific effects of interest. We considered model parameters as "non-null" when the 95% highest posterior density intervals (HPDI) excluded zero. HPDI is a Bayesian analogue of the frequentist confidence intervals.

1.1 Confirmatory Analysis

Our primary hypothesis regarded the two-way interaction between n-back level and the congruency effect. Both factors were within-participants: n-back Load (0-back, 1-back, 2-back, and 3-back; 2-back was the baseline level, as indicated in the preregistration) and Congruency (flanker stimuli:

congruent vs. incongruent flankers; congruent was the baseline level). The dependent variable was RT (conditional on accuracy). Accuracy of responses was also examined in a separate analysis.

As data consisted of a series of repeated individual responses (i.e., each participant provided hundreds of responses), the analysis was performed by fitting generalized linear mixed-effects models (GLMM). Congruency, Load, and their interaction were entered as the fixed effects of interest. Participants were entered as random effects in all models. We fitted a series of models with random intercepts by participants, and another series with also random slopes. As in the latter case convergence was more difficult to reach and the posterior distributions were practically the same in both cases, we eventually set only random intercepts in the reported final models. Convergence of the parameter estimates was assessed with the 'Rhat' (potential scale reduction factor on split chains). Rhat was below 1.01 for all parameters, indicating good convergence (at convergence, Rhat = 1.00).

RTs were modelled with the Gamma distribution and the Log link function. This assumes that RTs vary on a logarithmic scale, with variance increasing with longer RTs. This reflects the actual distribution of the data (see Table 1), and it is generally true for RTs. However, this represents a potential deviation from the preregistration document where the assumption concerning the distribution of RTs data was not discussed, but could be interpreted as tacitly suggesting the normal (Gaussian) distribution. Therefore, to ensure that the interpretation of the results was not bound to the assumptions on Gamma/Gaussian distribution of RTs, we subsequently repeated all analyses using the classical linear mixed-effects models (LMM, with the identity link function). This additional analysis is reported in the present Supplemental materials, Section 3. Finally, accuracy was modelled using a logistic regression, due to the inherently binomial (i.e., series of correct/incorrect responses) nature of this dependent variable.

1.2 Definition of Prior Knowledge

A set of informed priors based on the meta-analysis of the relevant previous literature were formalized. Prior formalization concerned the model parameters of the interaction of interest. This was limited to RT, as it is the dependent variable for which a load by congruency interaction was expected and theoretically motivated.

The interaction parameters define how the congruency effect varies in the alternative levels (0-, 1-, and 3-back) as compared to the baseline level (2-back). Positive values indicate larger congruency effect (i.e., expectedly the case of 0- and 1-back), and negative values indicate smaller congruency effect (i.e., expectedly in 3-back), as compared to the congruency effect estimated in the baseline level.

Five previous studies, which had an experimental design that closely resembled the current study, were selected (Berti and Schröger, 2003; Güldenpenning et al., 2020; Guerreiro et al., 2013; SanMiguel et al., 2008; Scharinger et al., 2015). For these prior studies, we coded the reported effects of interest with reference to a simple distinction between "low" and "high" load condition. In other words, we coded how the congruency effect differed between the "low" and "high" load condition. This contrast is thus expressed with one single value (i.e., a difference between differences). This value was assumed to represent plausible prior information for our parameter indicating the difference in congruency effect both in 1-back vs 2-back and 0-back vs 2-back. In practice, we assumed 0- and 1-back to be analogous to "low" load, and 2-back to be analogous to "high" load. Regarding 3-back, we assumed that it could represent a higher load condition than 2-back resulting in about 50% of the difference between 0-/1-back vs 2-back. However, this was only a guess, thus a very large uncertainty was placed on the prior for this parameter.

The prior formalization was performed using meta-analytic techniques, generally following the guidelines indicated by Borenstein, Hedges, Higgins, and Rothstein (2009). The "metafor" package (Viechtbauer, 2010) of the R software was used to perform the meta-analysis. Random effects

models were used for computing the meta-analytic estimates of interest (i.e., the interaction parameters). Random effects models were preferred to keep into account expectably large heterogeneity across studies (Borenstein et al., 2011).

Prior information was derived from descriptive statistics reported either in tables or in figures. In some cases, standard deviations had to be approximated from the standard errors of the means reported in the figures. With the sample size, means and standard deviations for both the congruent and incongruent condition at all load levels, and interaction parameters for each previous effect in each study could be estimated. For each effect, we performed a Monte Carlo simulation (10000 iterations) to estimate the most likely interaction parameter and its standard error, calculated respectively as the mean and standard deviation of simulated distributions. As our parameters had to be formalized for a GLMM with the Gamma family, we simulated averaged RT data that fitted with the Gamma distribution. At each iteration, N (equal to sample size) observations were drawn from a Gamma distribution having the mean and standard deviation equal to those reported as descriptive statistics. To do so, we simulated with coefficients shape = M2/SD2, rate = M/SD2 (with M and SD being those reported as the descriptive statistics in the paper). Once the simulated samples were drawn for all four values (2 Load x 2 Congruency) of interest, a generalized linear model (GLM) with the Gamma family, including the Load x Congruency interaction, was fitted on them, and the interaction parameter was extracted. In order to maximize prior uncertainty, data was simulated as if the four conditions of Load x Congruency were independent samples. We conservatively opted for an estimated prior distribution with higher uncertainty.

The Student's t distribution with 3 degrees of freedom was used to define prior distributions. Although similar in shape to the Normal distribution, it has substantially heavier tails, thus allowing for larger deviation from the prior norm. The mean of the prior distribution was set as the meta-analytic estimate, whereas its standard deviation was set as the standard error of the meta-analysis. As mentioned previously, the interaction parameter expressing the difference in congruency effect in the 3-back load level vs the 2-back load level was expected to be around 50% the effect formalized for "high" vs "low" load, because both 2-back and 3-back are high load levels, and the difference between the two may therefore be attenuated. However, as already emphasized, this was merely a speculation. Therefore, the standard deviation for the prior distribution of this parameter was set as five times larger than for the other parameters, thus making this prior only weakly informed.

1.3 Exploratory Analysis

In a follow-up analysis, we also examined the role of WMC as a potential moderator of the load by congruency interaction, to assess how this two-way interaction alters after adjusting for the continuous covariates of Ospan score and auditory digit span. Consideration of models' contrasts will be conditional to finding relevant interactions for their respective effects. As stated in the preregistration, along with the rationale for performing it, this analysis has to be considered exploratory as we did not expect it to have enough statistical power for assessing this possible interaction.

2 2 Formalization of the (G)LMM on Response Times

In this section we provide formal details on the statistical model fitted to predict the response times. Since in the manuscript we focused on the population-level effects, here we present details on the fixed part of the model.

2.1 Linear model

The linear part of the equation below shows how the expected mean values (μ_i) of RTs are calculated. Since the independent variables are factors (Congruency and Load), dummy coding is used.

$$\mu_i = \beta_0 + \beta_1 d^I_{1i} + \beta_2 d^I_{2i} + \beta_3 d^I_{3i} + \beta_4 d^{II}_{1i} + \beta_5 d^l_{1i} d^{II}_{1i} + \beta_6 d^l_{2i} d^{II}_{1i} + \beta_7 d^l_{3i} d^{II}_{1i}$$

where:

- β_0 represents the model intercept.
- β_1 to β_7 represent the other model coefficients.
- i in [1,8] indexes the eight estimated point values given by the 4 (Load: 0-back, 1-back, 2-back, 3-back) x 2 (Congruency: congruent vs incongruent) experimental design (see combinations below in Table 1).
- d^I_1, d^I_2, d^I_3 represent the dummy variables that refer to the Load factor; d^I_1 represent the dummy variable that refer to the Congruency factor; the full dummy coding scheme is summarized in Table 1

Table 1: Descriptive statistics. Means and standard deviations of self-turn error according to experimental conditions

i	**Load**	**Congruency**	**Dummy variable**			
			d^I_1	d^I_2	d^I_3	d^I_4
1	2-back*	Congruent	0	0	0	0
2	0-back	Congruent	1	0	0	0
3	1-back	Congruent	0	1	0	0
4	3-back	Congruent	0	0	1	0
5	2-back*	Incongruent	0	0	0	1
6	0-back	Incongruent	1	0	0	1
7	1-back	Incongruent	0	1	0	1
8	3-back	Incongruent	0	0	1	1

Note: *Levels marked with the asterisk represent the baseline levels in their respective factor.

2.2 Link function

The linear part of the model is the same for both LMM and GLMM. In the case of the GLMM, a link function is used. Specifically, the expected mean values (μ_i) are modelled by STAN via the following inverse link function:

$$\eta_i = \alpha + exp(-\mu_i)$$

and the gamma probability density function:

$$Gamma(y|\alpha, \eta_i) = \frac{\eta_i^\alpha}{\Gamma(\alpha)} * y^{\alpha-1} * exp(-\eta_i * y)$$

where α represents the shape parameter of the gamma distribution.

3 Individual-level Congruency Effects (analysis of random effects)

The following figures show, separately for each Load level, how the congruency effect varies across participants. Congruency is the estimated difference in RT between incongruent (supposedly slower) and congruent (supposedly faster) conditions. Estimates were calculated from a Bayesian GLMM with Gamma family using uninformed default priors (as the one presented in the manuscript), with random slopes for participants. Estimates of effect by participants are calculated from the random slopes. It is worth highlighting the consistency and similarity of the pattern for 0 and 1 back, whereby most participants showed a congruency effect and the average pattern was almost indistinguishable. This further confirms that the low cognitive load characterizing these conditions left open the possibility for the irrelevant auditory information to distract the vast majority of participants. At 3-back there is no longer evidence of the presence of the congruency effect at group level. Some individuals seem to show a reliable presence of the congruency effect and some others the opposite effect, as happens in null conditions. This further confirms that the high cognitive load characterizing these conditions did not allow the irrelevant auditory information to interfere. At 2-back performance was somehow intermediate.

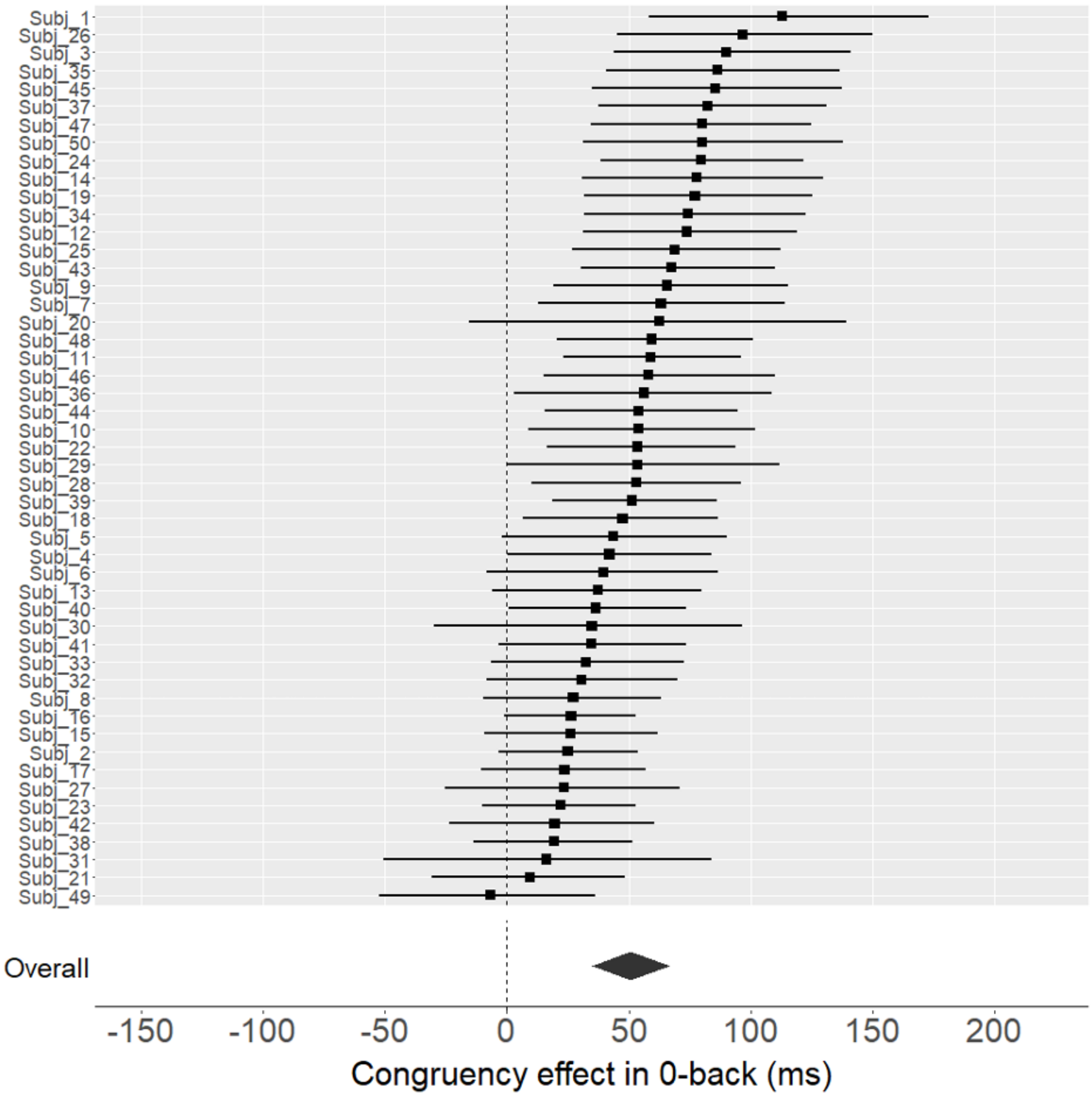

Figure 1: Individual congruency effect in 0-back (ms), ordered by decreasing estimated effect (top to bottom). *Note:* Error bars represent 95% Bayesian credible intervals (BCI) calculated with the percentile method. The black rhombus above the X-axis represents the estimated population-level effect with its 95% BCI.

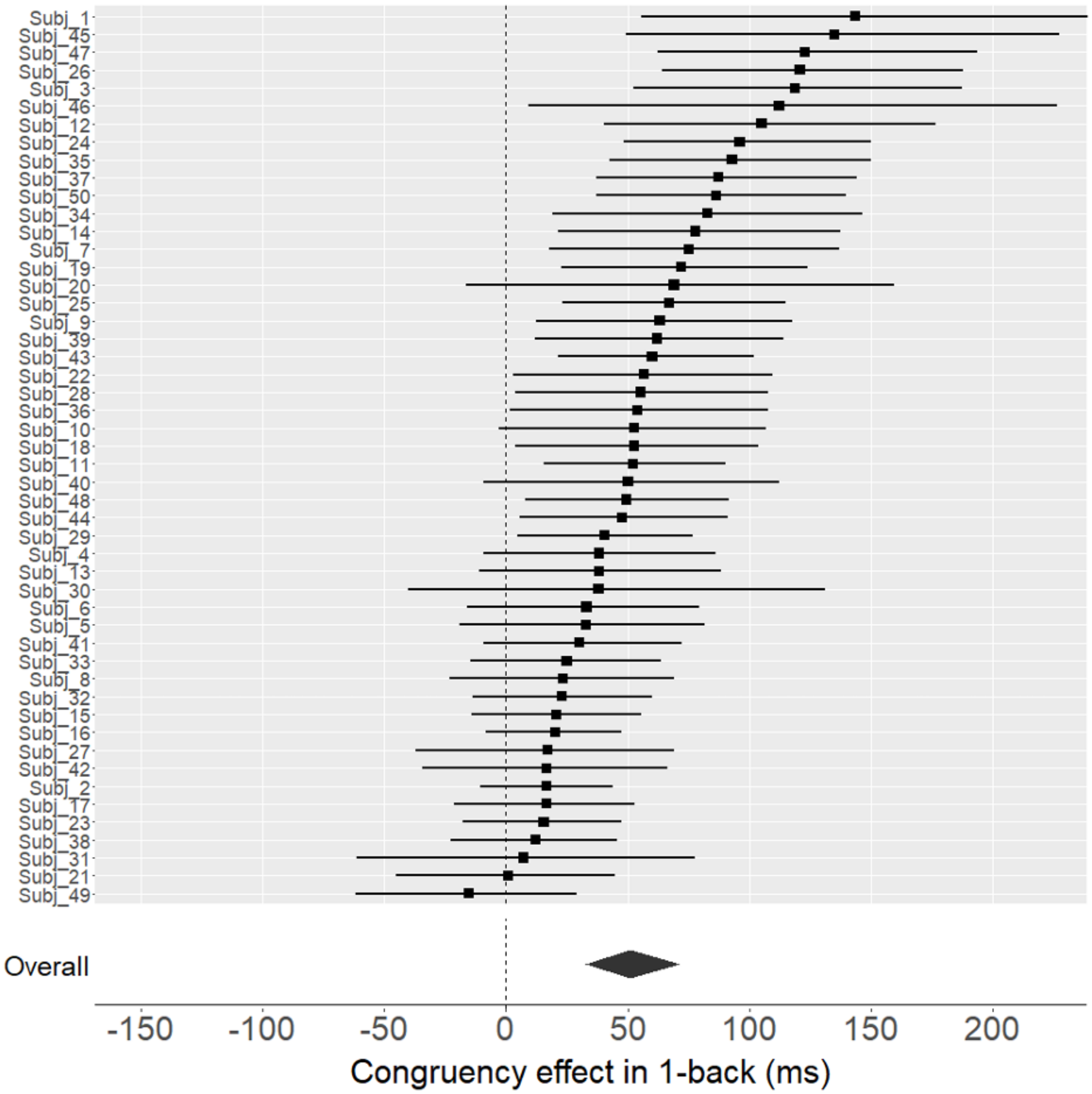

Figure 2: Individual congruency effect in 1-back (ms), ordered by decreasing estimated effect (top to bottom).*Note:* Error bars represent 95% Bayesian credible intervals (BCI) calculated with the percentile method. The black rhombus above the X-axis represents the estimated population-level effect with its 95% BCI.

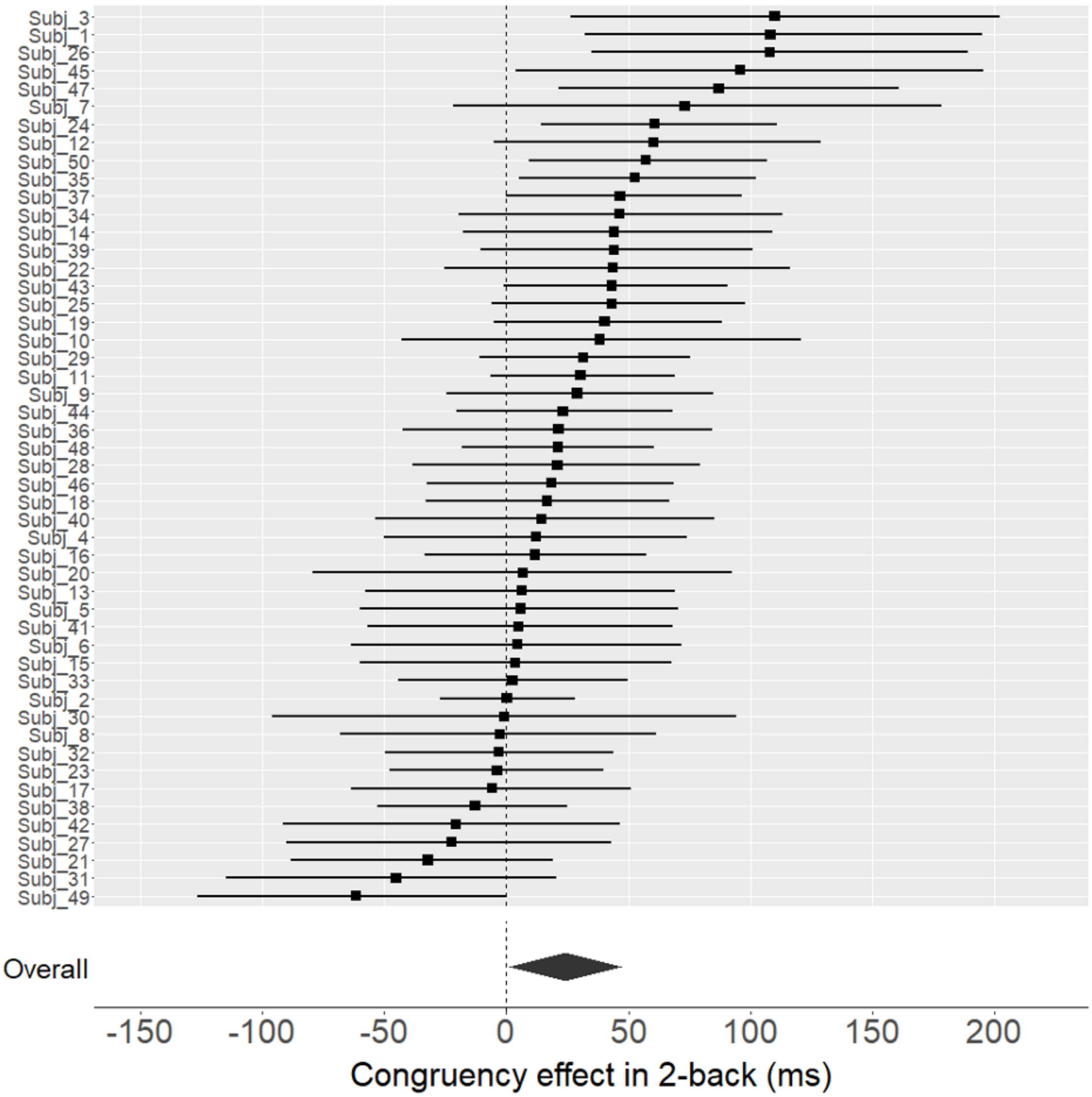

Figure 3: Individual congruency effect in 2-back (ms), ordered by decreasing estimated effect (top to bottom).*Note:* Error bars represent 95% Bayesian credible intervals (BCI) calculated with the percentile method. The black rhombus above the X-axis represents the estimated population-level effect with its 95% BCI.

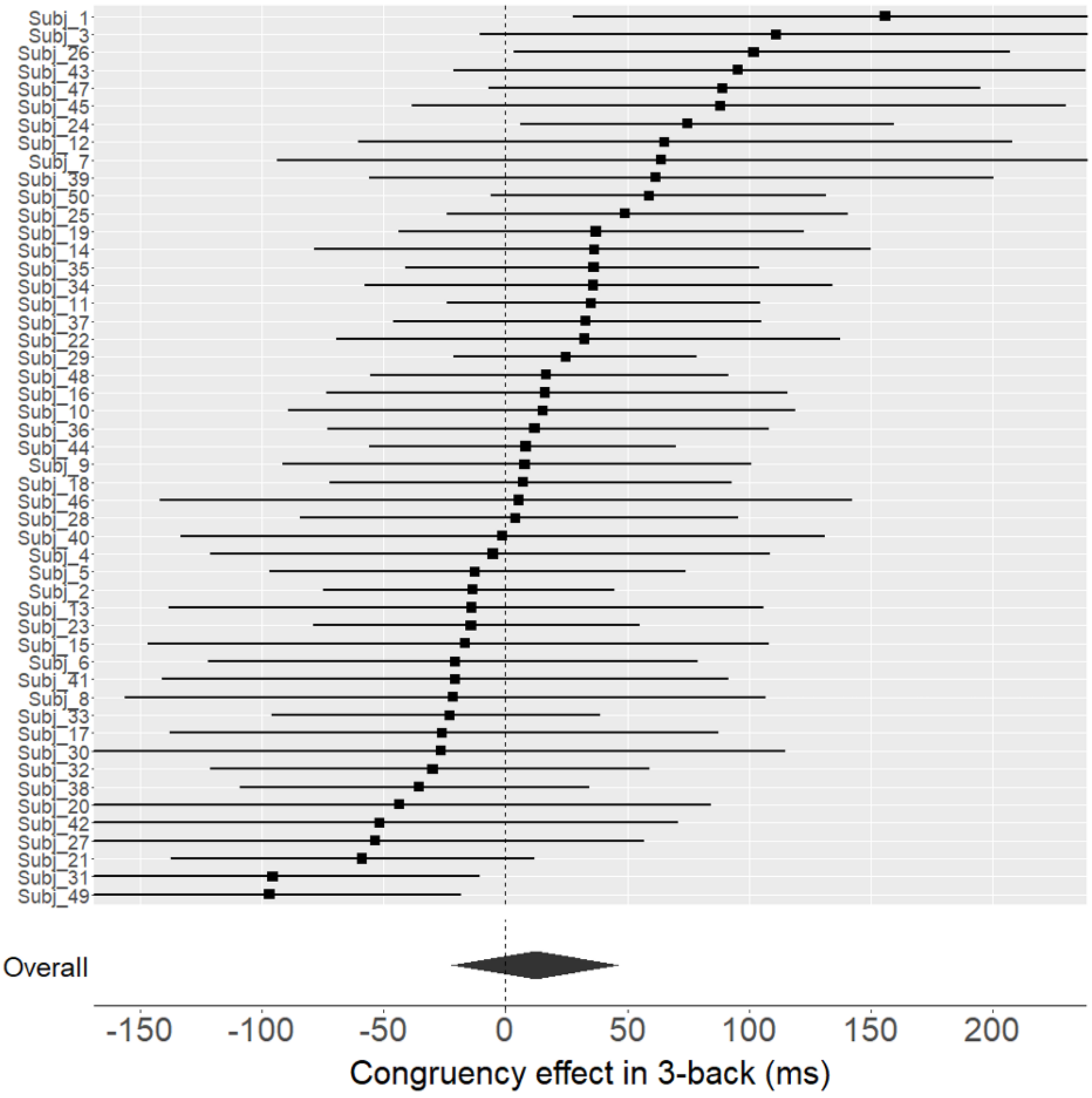

Figure 4: Individual congruency effect in 3-back (ms), ordered by decreasing estimated effect (top to bottom). *Note:* Error bars represent 95% Bayesian credible intervals (BCI) calculated with the percentile method. The black rhombus above the X-axis represents the estimated population-level effect with its 95% BCI.

4 Results Obtained Using LMM (i.e., with normality assumption) on RTs

This section focuses on confirmatory analysis of RTs when LMM (instead of GLMM with Gamma family, which is reported in the manuscript) were used. The estimated LMM parameters are reported below in Table 2. Unsurprisingly, LMM violated the normality assumption, as the residuals of the model reported below had skewness = 1.07 (for a standard normal, skewness = 0.00) and kurtosis = 5.30 (this is raw Pearson's kurtosis; for a standard normal, kurtosis = 3.00). This confirmed the appropriateness of using GLMM in the main analysis in the manuscript.

The 4 x 2 interaction between Load and Congruency was supported by strong evidence according to BF $>$ 1,000, or only moderate evidence according to more conservative WAIC: ΔWAIC = 4.2, ER = 8.17. Both main effects of Load and Congruency were supported by strong evidence: for both, BF $>$ 1,000 and ER $>$ 1,000 (ΔWAICs were 5,512.2 and 51.3 respectively). The estimated average RTs by load and congruency are shown, along with their credible intervals, in Figure 5. As can be seen, there seems to be a more pronounced congruency effect (i.e., difference in average RT between congruent and incongruent condition) in the 0-back and 1-back load level than in either 2-back or 3-back load level.

The evidence in favor of the interaction seemed to reflect statistical power (thanks to good sample size, effects examined at the within-participant level, and the analysis of all observations via mixed-effects linear model, which allowed to avoid any averaging) rather than the size of the effect of interest. The raw effect sizes, in terms of the average difference in RTs between congruent and incongruent condition at different load levels, were estimated from the posterior distributions of the model parameters, and were as follows: at 0-back, d = 49.89 ms, 95% HPDI (31.36, 68.86); at 1-back, d = 54.19 ms, 95% HPDI (34.49, 73.05); at 2-back, d = 26.23 ms, 95% HPDI (6.69, 46.52); at 3-back, d = 11.44 ms, 95% HPDI (-11.33, 34.89). Although these estimates were calculated from a different statistical model (LMM instead of GLMM) they present only minor variations from those reported in the manuscript.

Translating the effect sizes into standardized differences (Cohen's d) posed the problem of denominator selection. In this task, inter-individual variability seems less prominent than intra-individual variability. The residual intra-individual variability could be estimated as the model Sigma (i.e., the residuals SD after controlling for load and congruency; as the model also controls for random intercepts, inter-individual variability is already excluded from Sigma). The estimated Sigma was 339.46 ms, 95% HPDI (335.99, 342.92). On the contrary, inter-individual variability, estimated as the SD of the random intercepts by participants was SD = 186.24, 95% HPDI (149.73, 228.03). The second case (i.e., considering only inter-individual variability), is more akin to what is generally done in the literature, as intra-individual variability is generally lost with averaging procedures.

When the intra-individual variability was used as the denominator in the Cohen's d formula, the standardized effect sizes appeared very small. At 0-back, Cohen's d = .15, 95% HPDI (.09, .20); at 1-back, Cohen's d = .16, 95% HPDI (.10, .22); at 2-back, Cohen's d = .08, 95% HPDI (.02, .14); at 3-back, Cohen's d = .03, 95% HPDI (-.03, .10). The difference in congruency effect between 0- and 1-back combined vs 2- and 3-back combined was just ΔCohen's d = .10, 95% HPDI (.04, .16).

When the inter-individual variability was used as the denominator in the Cohen's d formula, the standardized effect sizes appeared slightly larger, albeit still limited. At 0-back, Cohen's d = .27, 95% HPDI (.16, .39); at 1-back, Cohen's d = .29, 95% HPDI (.18, .42); at 2-back, Cohen's d = .14, 95% HPDI (.03, .26); at 3-back, Cohen's d = .06, 95% HPDI (-.06, .19). The difference in congruency effect between 0- and 1-back combined vs 2- and 3-back combined was just ΔCohen's d = .18, 95% HPDI (.07, .31).

Note that this figure is analogous to Figure 1 in the manuscript, which shows the values estimated from the corresponding model using LMM with the Gaussian family. However, unlike the model using the Gaussian family, the one using the Gamma family allows for larger standard deviation – and thus larger uncertainty – for higher mean values, as reflected by the increasingly large error bars.

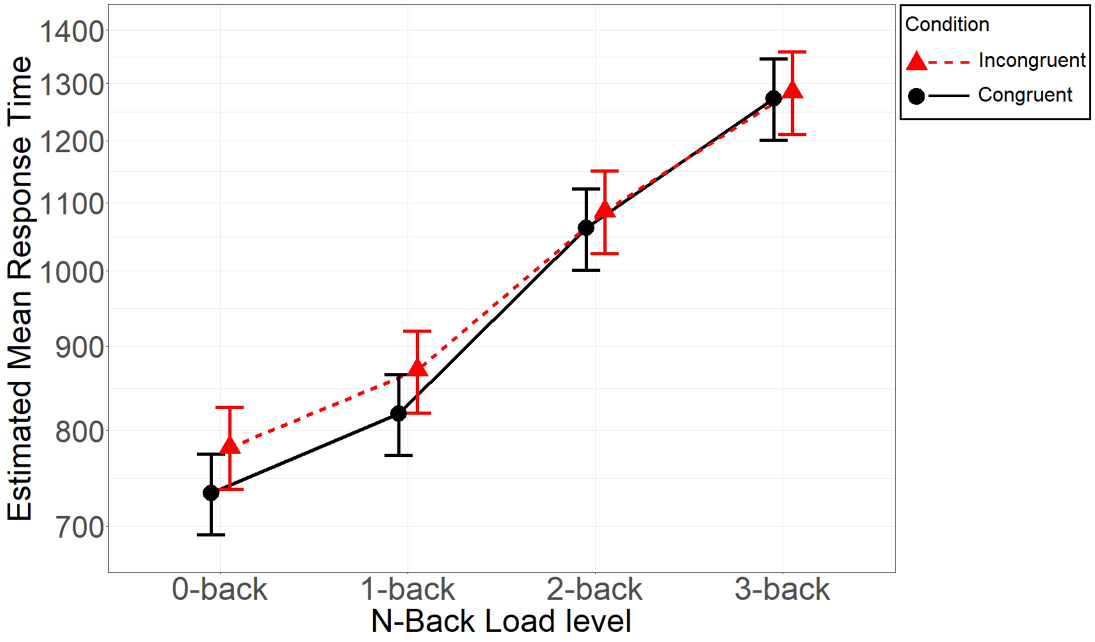

Figure 5: Estimated mean RT as a function of the Load by Congruency interaction, from the GLMM using the Gamma distribution for modelling RT. *Note:* Error bars represent 95% Bayesian credible intervals of the posterior estimates calculated with the percentile method.

Details on the prior distributions obtained in this analysis are reported in Table 2 . Estimate represents the mean value of the posterior distribution; SE represents its standard deviation. Baseline levels were “2-back” for Load and “congruent” for Congruency. The model was fitted using uninformed default priors. HPDI = Highest Posterior Density Interval. LMM = linear mixed-effects model.

Finally, with regards to the exploratory analysis on the potential moderating role of WMC on the Load x Congruency interactions, results were as follows. Concerning the digit span, evidence was against the 3-way interaction of interest, as it favored the model excluding it, ΔWAIC = -6.5, ER = .04. There was no evidence for a main effect of digit span either: when it was removed from the model including the load x congruency interaction, ΔWAIC = -0.6, ER = .74. Similarly, there was evidence against a 3-way interaction involving partial operation span as moderator of the Load x Congruency interaction, ΔWAIC = -3.4, ER = .18. There was no evidence for a main effect of partial operation span either, ΔWAIC = -0.7, ER = .70.

Table 2: Details on the posterior distributions of model parameters using LMM.

Response variable / Model coefficient	Estimate	SE	95%HDPI
**Response time (GLMM with Gaussian family)**			
Intercept	6.97	.03	(6.90, 7.02)
B - Load: 0-back	-.37	.01	(-.39, -.36)
B - Load: 1-back	-.26	.01	(-.27, -.24)
B - Load: 3-back	.18	.01	(.16, .20)
B - Congruency: Incongruent	.02	.01	(.00, .04)
B - Load x Congruency: 0-back	.04	.01	(.02, .07)
B - Load x Congruency: 1-back	.04	.01	(.01, .06)
B - Load x Congruency: 3-back	-.01	.01	(-.04, .01)
Shape	9.28	.10	(9.63, 10.02)

Note: These models were fitted by using default priors.

5 Prior Knowledge Formalization for the LMM on RTs and its Impact on the Posterior Distributions

As in the manuscript, we once again formalized prior knowledge concerning the interaction parameters of interest, now for the LMM on RTs. The same five previous studies were taken into consideration. These were: Berti and Schröger (2003), Guerreiro et al. (2013), Güldenpenning et al. (2020), SanMiguel et al. (2008), Scharinger et al. (2015).

The interaction parameters of interest were those reported in Table 1 in the manuscript: : "Load x Congruency: 0-back", "Load x Congruency: 1-back", and "Load x Congruency: 3-back". This represents how much larger (or smaller) the congruency effect is in the alternative levels (0-, 1-, and 3-back) as compared to the baseline level (2-back). Positive values indicate larger congruency effect (as in the case of 0- and 1-back), and negative values indicate smaller congruency effect (as in 3-back), as compared to the congruency effect estimated in the baseline level.

With the sample size, mean and (estimated) standard deviations for both the congruent and incongruent condition at all Load levels, we could estimate the interaction parameters. As described in the manuscript, for each reported effect, we performed a Monte Carlo simulation to estimate the most likely interaction parameter and its standard error, calculated respectively as the mean and standard deviation of simulated distributions. We ran 5,000 iterations. At each iteration, N (equal to sample size) observations were drawn from a normal distribution having the mean and standard deviation equal to those reported as the descriptive statistics. Once the simulated samples were obtained for all four values (2 Load x 2 Congruency) of interest, a linear

model (LM) including the load x congruency interaction was fitted on them, and the interaction parameter was extracted ("high load" was set as the baseline level).

The forest plots showing the estimated interaction parameters of interest for each study, as well as the meta-analytic estimates obtained with a random effects model, are shown in Figure 6. The prior distributions were formalized as reported in Table 3.

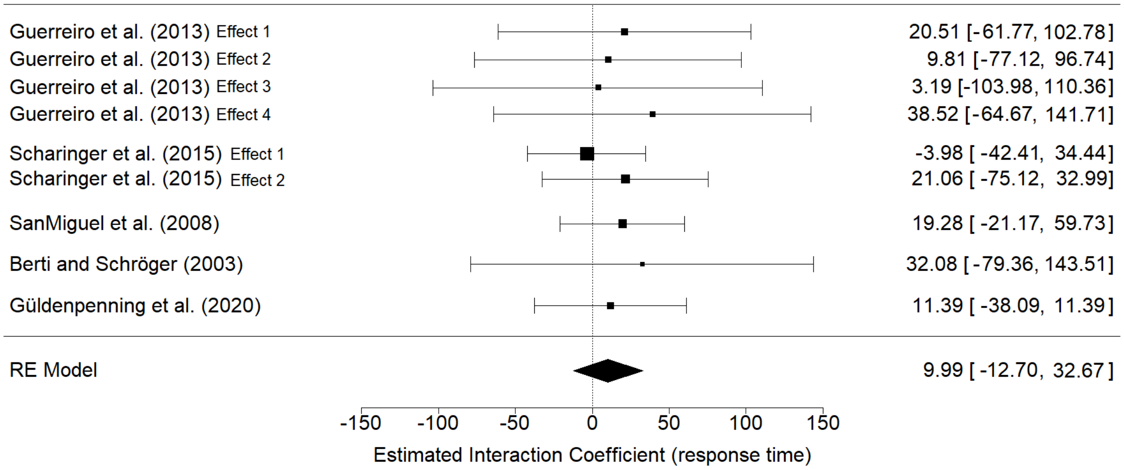

Figure 6: Individual congruency effect in 0-back (ms), ordered by decreasing estimated effect (top to bottom).

Table 3: Prior distributions formalized for the interaction parameters of interest.

**Interaction parameter** / **Family of the model**	**Distribution: Student's t with 3 degrees of freedom** M	SD
**LMM with Gaussian family**		
B - Load x Congruency: 0-back	9.99	11.57
B - Load x Congruency: 1-back	9.99	11.57
B - Load x Congruency: 3-back	-5.00	57.87

Note: The baseline level for "load" is 2-back; "congruent" for congruency.

Compared to the GLMM with Gamma family presented in the manuscript, the new LMM model embedding the informed priors provided very similar point estimates. Nonetheless, in this case the prior expectations slightly diverged from the likelihood of our data, in the sense that the

meta-analysis suggested effect sizes that were smaller than those observed in our data, see Figure 7. Specifically, it was calculated that the difference in congruency effect between low load (equivalent to our 0- and 1-back) and high load (equivalent to our 2- and 3-back) would be around $\Delta d = 10.00$ ms, whereas the observed effects were larger. See Figure 5 and Table 2 for the estimated parameters using uninformed default priors (i.e., parameters estimated solely based on the data) as well as using informed priors. This further confirms that fitting GLMM using the Gamma family, as opted for in the manuscript, was more appropriate than the use of LMM as described here.

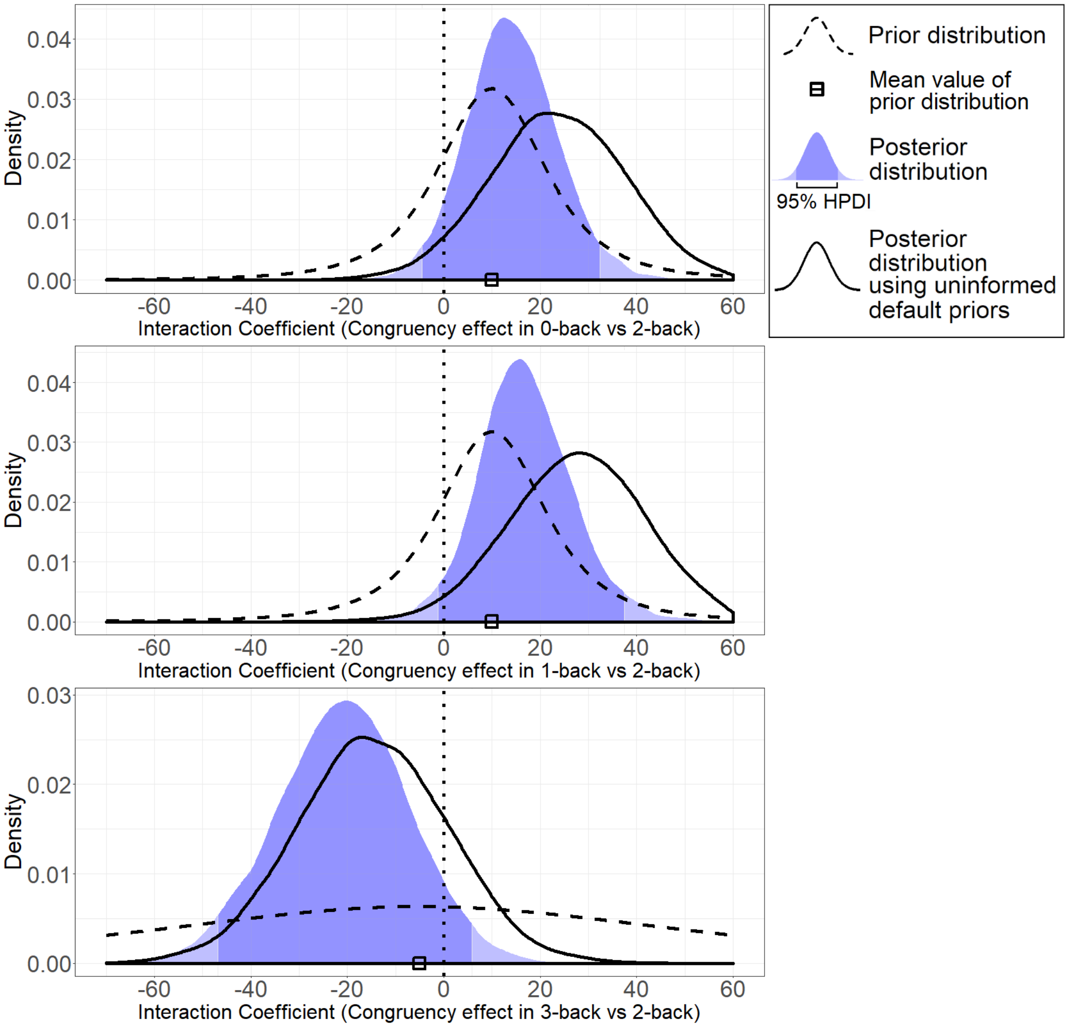

Figure 7: Prior and posterior distributions for the three interaction parameters on RTs.

References

Berti, S., & Schröger, E. (2003). Working memory controls involuntary attention switching: Evidence from an auditory distraction paradigm. *European Journal of Neuroscience*, *17*(5), 1119–1122. https://doi.org/10.1046/j.1460-9568.2003.02527.x

Borenstein, M., Hedges, L. V., Higgins, J. P., & Rothstein, H. R. (2011). *Introduction to meta-analysis*. John Wiley & Sons.

Bürkner, P.-C. Et al. (2017). Brms: An r package for bayesian multilevel models using stan. *Journal of statistical software*, *80*(1), 1–28. https://doi.org/10.18637/jss.v080.i01

Burnham, K. P., Anderson, D. R., & Huyvaert, K. P. (2011). Aic model selection and multimodel inference in behavioral ecology: Some background, observations, and comparisons. *Behavioral ecology and sociobiology*, *65*(1), 23–35. https://doi.org/10.1007/s00265-010-1029-6

Guerreiro, M. J., Murphy, D. R., & Van Gerven, P. W. (2013). Making sense of age-related distractibility: The critical role of sensory modality. *Acta psychologica*, *142*(2), 184–194. https://doi.org/10.1016/j.actpsy.2012.11.007

Güldenpenning, I., Kunde, W., & Weigelt, M. (2020). Cognitive load reduces interference by head fakes in basketball. *Acta Psychologica*, *203*, 103013. https://doi.org/10.1016/j.actpsy.2020.103013

McElreath, R. (2016). *Statistical rethinking: A Bayesian course with examples in R*. Chapman; Hall/CRC. https://doi.org/10.1201/9781315372495

R Core Team. (2019). R: A language and environment for statistical computing. *Vienna, Austria.* https://www.R-project.org/

SanMiguel, I., Corral, M.-J., & Escera, C. (2008). When loading working memory reduces distraction: Behavioral and electrophysiological evidence from an auditory-visual distraction paradigm. *Journal of Cognitive Neuroscience*, *20*(7), 1131–1145. https://doi.org/10.1162/jocn.2008.20078

Scharinger, C., Soutschek, A., Schubert, T., & Gerjets, P. (2015). When flanker meets the n-back: What eeg and pupil dilation data reveal about the interplay between the two central-

executive working memory functions inhibition and updating. *Psychophysiology*, *52*(10), 1293–1304. https://doi.org/10.1111/psyp.12500

Schönbrodt, F. D., Wagenmakers, E.-J., Zehetleitner, M., & Perugini, M. (2017). Sequential hypothesis testing with bayes factors: Efficiently testing mean differences. *Psychological methods*, *22*(2), 322. https://doi.org/10.1037/met0000061

Viechtbauer, W. (2010). Conducting meta-analyses in r with the metafor package. *Journal of statistical software*, *36*(3), 1–48. https://doi.org/10.18637/jss.v036.i03

Wagenmakers, E.-J., & Farrell, S. (2004). Aic model selection using akaike weights. *Psychonomic bulletin & review*, *11*(1), 192–196. https://doi.org/10.3758/BF03206482
